# Synthesis and Characterization of New Organic Dyes Containing the Indigo Core

**DOI:** 10.3390/molecules25153377

**Published:** 2020-07-25

**Authors:** Daniele Franchi, Massimo Calamante, Carmen Coppola, Alessandro Mordini, Gianna Reginato, Adalgisa Sinicropi, Lorenzo Zani

**Affiliations:** 1 Dipartimento di Chimica “Ugo Schiff”, Università degli Studi di Firenze, Via della Lastruccia, 13, 50019 Sesto Fiorentino, Italy; daniele.franchi87@gmail.com (D.F.); mcalamante@iccom.cnr.it (M.C.); 2 CNR-Istituto di Chimica dei Composti Organometallici (CNR-ICCOM), Via Madonna del Piano, 10, 50019 Sesto Fiorentino, Italy; adalgisa.sinicropi@unisi.it (A.S.); lorenzo.zani@iccom.cnr.it (L.Z.); 3 Department of Chemistry, KTH, Teknikringen 30, 10044 Stockholm, Sweden; 4 R2ES Lab, Dipartimento di Biotecnologie, Chimica e Farmacia, Università degli Studi di Siena, Via A. Moro, 2, 53100 Siena, Italy; carmen.coppola@student.unisi.it; 5 CSGI, Consorzio per lo Sviluppo dei Sistemi a Grande Interfase, Via della Lastruccia, 3, 50019 Sesto Fiorentino, Italy

**Keywords:** indigo dyes, DSSC, synthesis, cross coupling, spectroscopy

## Abstract

A new series of symmetrical organic dyes containing an indigo central core decorated with different electron donor groups have been prepared, starting from Tyrian Purple and using the Pd-catalyzed Stille-Migita coupling process. The effect of substituents on the spectroscopic properties of the dyes has been investigated theoretically and experimentally. In general, all dyes presented intense light absorption bands, both in the blue and red regions of the visible spectrum, conferring them a bright green color in solution. Using the same approach, an asymmetrically substituted D–A-π–A green dye, bearing a triarylamine electron donor and the cyanoacrylate acceptor/anchoring group, has been synthesized for the first time and fully characterized, confirming that spectroscopic and electrochemical properties are consistent with a possible application in dye-sensitized solar cells (DSSC).

## 1. Introduction

**Figure 1:**
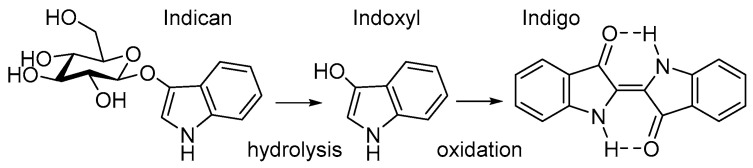
Biosynthesis of Indigo from Indican.

Indigo (also known as C.I. Vat Blue 1) is a naturally occurring blue dye, originally obtained by extraction of indican from plants. Acid-hydrolysis and mild oxidation produce the dye (Figure 1), which has been known since ancient times and in different civilizations. Since the beginning, due to the shortage of natural blue dyes, indigo has played an important role in the economies of many countries, being mainly used for textile dyeing and printing and, indeed, it is still used in the fabric industry today, where it has probably the largest application in denim [1].

**Figure 2:**
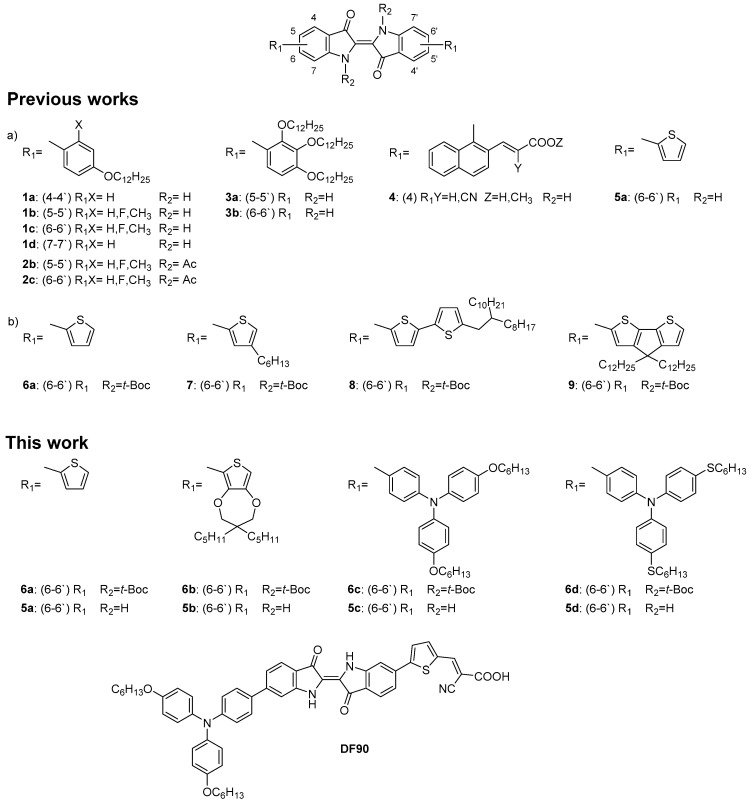
Conjugated indigo derivatives reported in the literature and dyes developed in this study. (**a**) non *t*-Boc-protected compounds; (**b**) *t*-Boc-protected compounds).

The first synthesis of indigo was reported by Adolf von Baeyer in 1882 [2] and its chemical structure was elucidated one year later [3]. Shortly after, a practical manufacturing process was developed and since 1897 natural indigo has almost been replaced by the synthetic molecule, which is probably the most produced dye in the world [4]. Concerning the spectroscopic properties of indigo [5,6,7,8,9], several studies have been reported, showing how the absorption spectrum of the dye is dependent on the environment, ranging from red (540 nm) in the gas phase, to violet (588 nm) in tetrachloromethane, to blue (606 nm) in polar solvents such as ethanol [8]. Moreover, indigo has extremely low solubility in water and in common organic solvents, a high melting point (390–392 °C), and gives highly-crystalline thin films upon evaporation. This behaviour is mainly due to the possibility to form inter- and intramolecular hydrogen bonds as well as strong intermolecular π-interactions, which are also responsible for providing very good charge transporting properties. Accordingly, indigo is an intrinsically ambipolar organic semiconductor with a bandgap of 1.7 eV, high and well-balanced electron and hole mobilities (approx. 1 × 10^−2^ cm^2^/V·s) and good stability against degradation in air [10]. For these reasons, the dye has been recently exploited for the application in the field of natural and sustainable semiconductors, aiming to tackle the problem of electronic waste by using naturally occurring, low toxic and biodegradable materials [11]. For instance, indigo and its derivatives have found application as semiconductors in field-effect transistors [4,10,11], sensors [12,13], electrodes for ion batteries [14,15] and liquid crystals [16,17]. Moreover, the natural dye extracted from *Indigofera tinctorial* [18] has been used to prepare dye-sensitized solar cells (DSSC), a novel class of photovoltaics which represent a promising alternative to traditional silicon-containing devices [19,20]. The working principle of a DSSC is inspired by natural photosynthesis, as the light harvest is carried out by a dye, which is absorbed on a thin layer of a mesoporous semiconductor (usually TiO_2_). For such an application some natural dyes have been used for titania sensitization, although low photocurrent conversion efficiencies (PCE) have been generally observed so far [21]. On the contrary, when specially designed molecules have been tested, better results were found, with record efficiency up to 13.6% [22]. In particular, donor-π-bridge-acceptor (D-π-A) structures [23,24,25] are conventionally used in the design of organic photosensitizers and, frequently, triphenylamine (TPA) and cyanoacrylic acid have been established to be optimal electron donor and electron acceptor substituents for obtaining efficient devices. Concerning the π-bridge, a large number of different heterocycles have been screened leading to several classes of sensitizers successfully used in DSSC. The vast majority of them, however, absorb light only in the blue and green regions of the spectrum (400–600 nm), giving rise to orange/red-colored devices, with the exception of those containing specific chromophores such as, for example, squaraines [26,27,28,29,30]. Blue and green dyes [31,32], on the other hand, have a high commercial interest due to both their lovely colours and their capability to absorb the incident photons also in the red and near-infrared region (NIR) of the spectrum (600–800 nm), maximizing solar light harvesting. A possible strategy to design this kind of sensitizers is that of introducing an additional acceptor unit between the donor and the conjugated bridge, modifying D-π-A structures into D-A-π-A ones. In this way, it is possible to affect the energy levels of the sensitizers, and maybe also improve their photostability [33,34]. Following this approach, the indigo unit can be considered a very interesting auxiliary acceptor to be inserted into a D-A-π-A structure with the aim of extending the absorption range and obtaining blue-green coloured organic sensitizers. However, despite theoretical design supported the possibility to use indigo derivatives as DSSC sensitizers [35,36]. D-A-π-A indigo-based dyes have never been prepared and tested for such an application. From the synthetic point of view, the modification of the pristine indigo to a more extended conjugated structure is not an easy task, due to its low solubility. As a matter of fact, the first indigo derivatives with conjugated aromatic substituents to be reported were obtained by modification of the precursors of the indigo core [16,17,37,38]. Procedures for the modification of preformed indigo are more recent and take advantage mainly of cross-coupling reactions of the 6-6′ dibromo derivative Tyrian Purple (C.I. Natural Violet) with electrophiles (Figure 2) [38,39,40,41]. Spectroscopic characterization of such derivatives pointed out that it is possible to affect the energy levels of indigoids by chemical design and that the effect of substituents can be qualitatively predicted by DFT calculations. Moreover, derivatization can drastically enhance the solubility in organic solvents, especially for functionalization in 4-4′ and 7-7′ position, resulting in twisting and buckling with respect to the central double bond [16].

In this paper, we report the preparation and the full spectroscopic characterization of some new indigo-based dyes (Figure 2). In particular, some symmetrical D-A-D dyes featuring an extended conjugation have been obtained, using a synthetic approach based on the Pd-catalyzed Stille-Migita coupling, which was performed in very mild conditions. The new dyes have been spectroscopically characterized and their optical properties compared with the results of computational investigations, in order to understand how their structure influences the interaction with light and evaluate the nature and energy of their electronic transitions. Based on these studies, an unsymmetrically substituted indigo-based D-A-π-A dye was then designed, synthesized, and fully characterized, to assess the possibility of using this scaffold to prepare blue-green dyes for DSSC application.

## 2. Results and Discussion

### 2.1. Computational Investigation

To extend the conjugation of the indigo scaffold we decided to exploit the effect of triarylamine- and thienyl groups, as they are very frequently used in the design of organic semiconductor materials as well as DSSC sensitizers. In particular, we considered four symmetrical dyes **5a**–**d** and a typical D-A-π-A structure such as **DF90** (Figure 2), where the cyanoacrylate moiety is essential not only as an acceptor group (facilitating electron injection in the conduction band of TiO_2_) but also to ensure the anchoring of the dye to the semiconductor surface.

The B3LYP/6-31G** optimized geometries, both in vacuo and in DCM, of keto–keto (KK), keto–enol (KE) and enol–enol (EE) tautomers of compounds **5a**, **5b**, **5c**, **5d**, and **DF90** display a planar structure in the central indigo scaffold (dihedral angles ≤0.2°), whereas a more pronounced torsion is observed for the bonds with R_1_ moieties (dihedral angles between 17° and 34°) (see Supporting Information Figures S1–S4, for **5d** only the KK tautomer is considered). No significant differences between the *in vacuo* and in DCM optimized geometrical parameters and relative energies are found for all investigated compounds (see Supporting Information Table S1). On the basis of the Boltzmann equation, the room temperature ΔG values, computed on two representative molecules **5a** and **DF90,** clearly indicate that the only species that would be present in solution is the KK tautomer (see Supporting Information Table S2). For such reason, in the following, we present and discuss only the results obtained for the KK tautomers. The complete set of data including KE and EE tautomers is reported in the Supporting Information.

**Figure 3:**
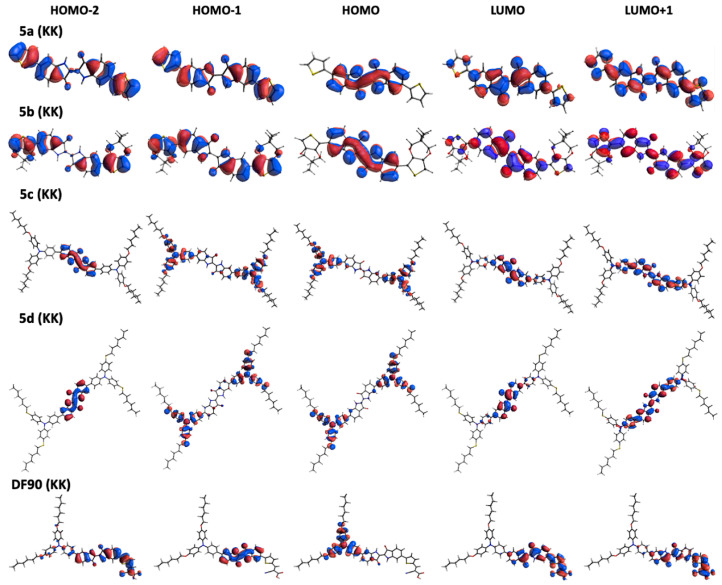
B3LYP/6-31G** ground-state electron density distributions in DCM of KK tautomer of compounds **5a**, **5b**, **5c**, **5d** and **DF90**.

The absorption maximum (λ^a^_max_), vertical excitation energy (E_exc_) and oscillator strengths (*f*) computed in DCM on the minimized structures of KK tautomer of compounds **5a**, **5b**, **5c**, **5d**, and **DF90** are shown in Table 1. The DFT frontier molecular orbitals (FMOs) of the transitions are shown in Figure 3.

Orbital plots are similar for compounds **5a** and **5b**: the HOMO and LUMO, i.e., the FMOs involved in the lowest energy transition predicted at 604–610 nm, are localized over the indigo moiety and have a π and π* character, respectively. This charge distribution is the same found for indigo and its derivatives in previous literature papers [9,38,42,43].

Similar localized orbitals correspond also to the HOMO-2 and LUMO of molecules **5c** and **5d**. Indeed, the absorption predicted at about 594–596 nm for **5c** and **5d** is due to the HOMO-2→LUMO transition. Likewise, the HOMO-1→LUMO transition of the asymmetric compound **DF90**, which corresponds to the absorption maximum at about 645 nm, is characterized by the same ground-state electron density delocalization on the Indigo part of the molecule. In this last case, the limited spatial separation between these frontier molecular orbitals suggests a consequent limited intramolecular charge separation upon photoexcitation of the dyes. The DFT FMOs energies obtained in DCM for tautomer KK of **5a**, **5b**, **5c**, **5d**, and **DF90** are reported in the Supporting Information (Figure S5).

### 2.2. Synthesis of Dyes

**Scheme 1:**
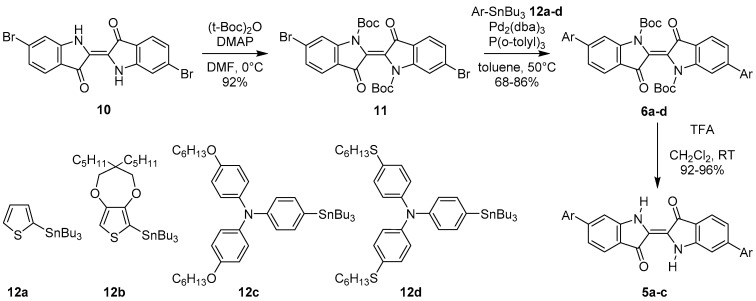
Stille-Migita cross-coupling of t-Boc-protected Tyrian Purple (**10**) with thienyl- and triarylamino-stannanes 8a**–**d.

To prepare the symmetrical dyes **5a**–**d** we used an approach (Scheme 1) similar to that already described for the synthesis of 6,6′-dithienylindigo [38]. Tyrian Purple (**10**) was obtained as a purple powder in 71% yield, using the classical indigo-forming protocol [2]. To increase its solubility and simplify its chemical manipulation and processing, Tyrian Purple was protected using (*t*-Boc)_2_O and DMAP in DMF solution, and the soluble deep red product **11** was obtained in 92% yield. In order to preserve the required but thermolabile *t*-Boc protection, the introduction of the side groups should occur under very mild and chemoselective conditions. For these reasons we decided to take advantage of the mild conditions usually applied in the Stille-Migita cross-coupling, and thus to react intermediate **11** with stannanes **12a**–**d** (Scheme 1).

We optimized the reaction conditions using commercially available stannane **12a** and found that Pd_2_(dba)_3_ and the electron-rich tri(*o*-tolyl)phosphine was the best combination to generate the active catalytic species able to perform the cross-coupling at 50 °C. In this way, deprotection of the *t*-Boc group, which might occur when temperatures higher than 90 °C are used [44,45] and the consequent precipitation of unprotected starting material was prevented. Thus, using toluene as solvent and two equivalents of stannane **12a**, symmetric indigo derivative **6a** was obtained in good yield in 5 h. The reaction was then repeated using the more electron-rich thienylstannane **12b**, and two triarylamine-containing stannanes **12c** and **12d**, which were prepared as previously reported. [44,46] In all cases, the corresponding coupling compounds **6b**–**d** were recovered with high yields after purification. Unfortunately, compound **6d** appeared unstable in solution ad could not be fully characterized nor used for further deprotection. However, we were able to identify it by ESI/MS and to record UV/visible spectra. Finally, reaction of **6a**–**c** with TFA occurred smoothly at room temperature to give compounds **5a**–**c**, which were soluble in the most common organic solvents**.**

**Scheme 2:**
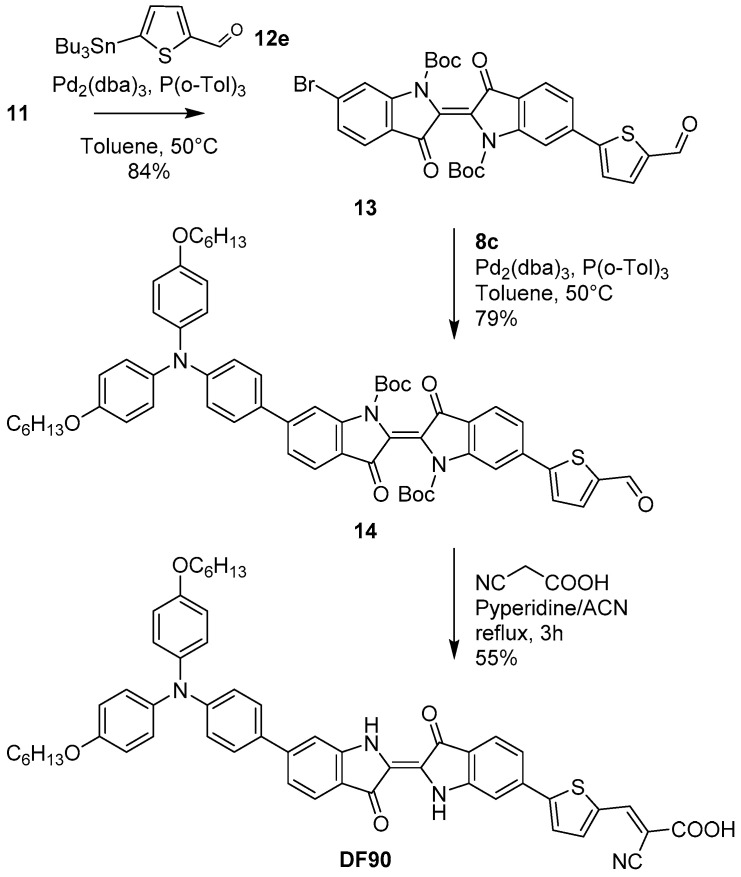
Synthesis of dye **DF90**.

To prepare dye **DF90** a modification of the above-described synthetic approach was necessary in order to obtain a non-symmetrical molecule. Again we used the *t*-Boc protected Tyrian Purple (**11**) as starting material and decided to install the acceptor moiety first (Scheme 2).

To this end, we prepared stannane **12e** [44] carrying a formyl group, which was essential to later establish the required cyanoacrylic moiety (Scheme 2). Desymmetrization of compound **11** with stannane **12e** needed to be carried out using a large excess (five-fold) of the starting material**,** which, opportunely, could be easily recovered at the end of reaction by precipitation from ethyl acetate/hexane mixture. Evaporation of the solvent gave intermediate **13** which was obtained in 84% yield (based on **12e**) after column chromatography. The second coupling, required to introduce the donor group, was performed in essentially the same conditions, albeit using one equivalent of stannane **12c**: pure *t*-Boc protected aldehyde **14** was thus obtained in 79% yield after purification. Knoevenagel condensation of aldehyde **14** with cyanoacetic acid and piperidine in acetonitrile allowed introduction of the desired cyanoacrylate together with the simultaneous deprotection of *t*-Boc groups, affording dye **DF90** in 55% yield. 

### 2.3. Optical and Electrochemical Properties

**Figure 4:**
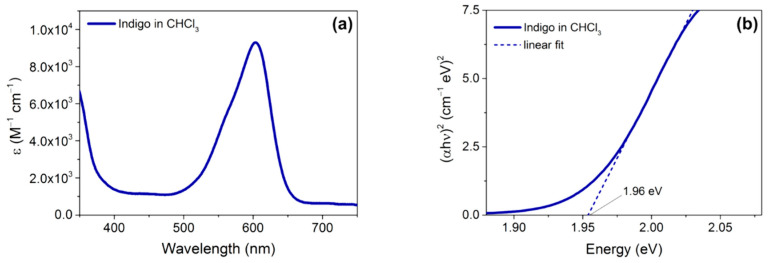
UV-Vis absorption spectrum of indigo dissolved in CHCl_3_ (**a**) and corresponding Tauc plot (**b**).

The optical properties of all the new dyes were studied and compared with those of the parent compound indigo (Figure 4). It must be considered, however, that when Indigo was suspended in CHCl_3_ in the reported conditions (0.17 g/mL corresponding to 6.5 × 10^−5^ M), it was not possible to obtain a completely clear solution, thus the resulting molar extinction coefficient must be taken only as an approximate value.

In good agreement with previous literature reports [47], we observed a relatively intense transition at 604 nm, corresponding to an *E_0-0_* of 1.96 eV, due to the so-called “*H*-chromophore” [8,38,48] corresponding to a cross-conjugated donor-acceptor system held together by intramolecular hydrogen bonds (see Figure 1). Computational studies revealed that the H-chromophore absorption is due to a HOMO-LUMO transition with π-π* character, corresponding to a net electron transfer from the N–H group (acting as a donor) to the C=O group (acting as an acceptor) [9]. The UV-vis spectra of dyes **5a**–**c** were recorded in CH_2_Cl_2_ and EtOH solution. Spectra are reported in Figure 4 and compared with those of the corresponding *t*-Boc protected compounds **6a**–**d**. Due to its low solubility in EtOH, the spectrum of compound **6b** could be recorded only in CH_2_Cl_2_. All relevant spectroscopic data have been summarized in Table 2.

The absorption maxima for the lowest energy band of **5a**–**5c** and **DF90** (see below) were well predicted by the results of the computational investigation. Indeed, the differences between DFT and experimental vertical excitation energies (E_exc_) were 0.05 eV at most. In absence of the experimental values for **5d**, we computed the geometry, orbital energies, and UV-Vis properties for compound **6d** (see Supporting Information, Figure S4, Tables S4 and S6). The presence of the *t*-Boc group causes a slight deviation from the planarity of the central C–C bond of the indigo moiety (dihedral angle changes from 0° to 5°) which in turns lead to a blue-shift of the lowest energy band.

**Figure 5:**
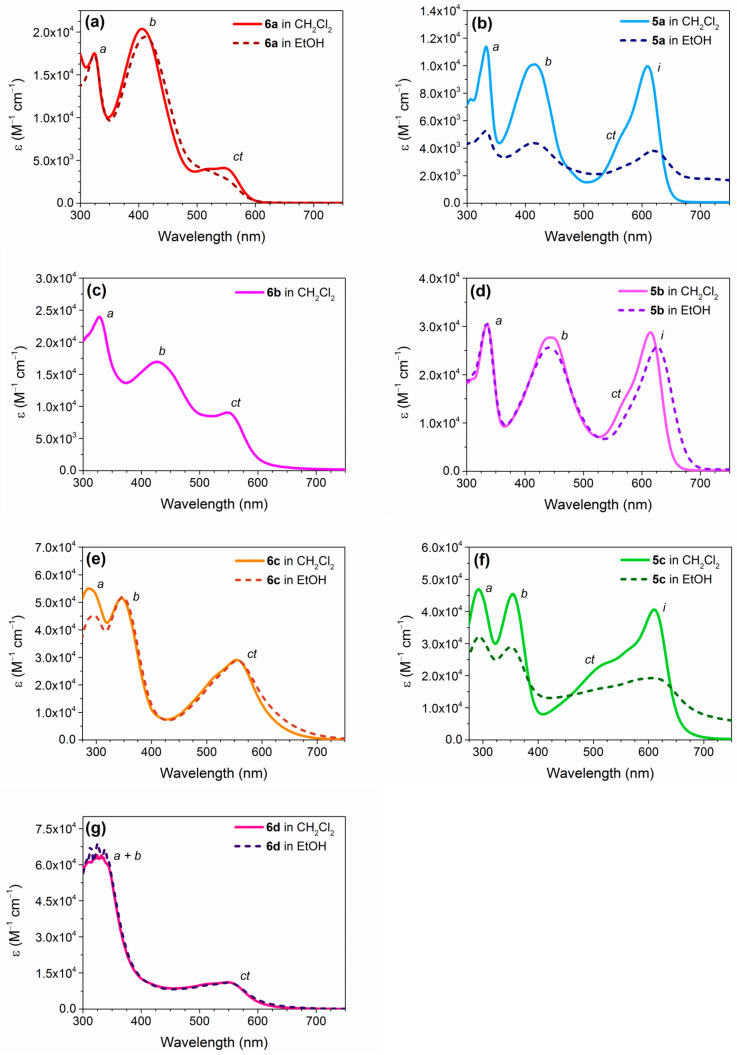
UV-Vis absorption spectra of dyes **6a–d** (panels (**a**), (**c**), (**e**), (**g**), respectively) and **5a–c** (panels (**b**), (**d**), (**f**), respectively).

Significant differences were observed between the *N*-*t*-Boc protected and free N–H species (Figure 5). Considering the *t*-Boc-protected compounds **6a**–**d**, while the first two peaks (marked as a,b) can be assigned to localized π-π* transitions involving different parts of the molecules, the lower energy band is likely due to a charge transfer (ct) transition between the lateral donor groups and the central acceptor unit: this hypothesis is supported by the fact that such band is most red-shifted and intense in the case of compound **6c**, featuring the strongly electron-donating hexyloxy-TPA side groups. As a consequence of the particular absorption profile of the dyes, the corresponding solutions appeared red to purple in color.

No significant difference was observed when passing from CH_2_Cl_2_ to EtOH, with all compounds displaying essentially the same spectra, highlighting also their good solubility in the more polar and protic solvent (except for **6b**). Moving from *N*-Boc protected compounds **6a**–**d** to free *N*–H compounds **5a**–**c** the main change observed in the absorption spectra was the activation of the “*H*-chromophore” transition (indicated as *i*). In analogy with the parent indigo compound, such transition, in CH_2_Cl_2_ solution, appeared as an intense peak in the 610–614 nm range, corresponding to *E_0-0_* values of 1.93–1.95 eV. The original *ct* band already observed for compounds **6a**–**d** was still present in the spectra of compounds **5a**–**c**, but appeared only as a shoulder of the more intense indigo transition. Furthermore, in analogy to its precursor **6c**, derivative **5c** had a relatively strong *ct* band, which together with the *i* transition gave rise to an intense panchromatic absorption in the 400–650 nm range.

**Figure 6:**
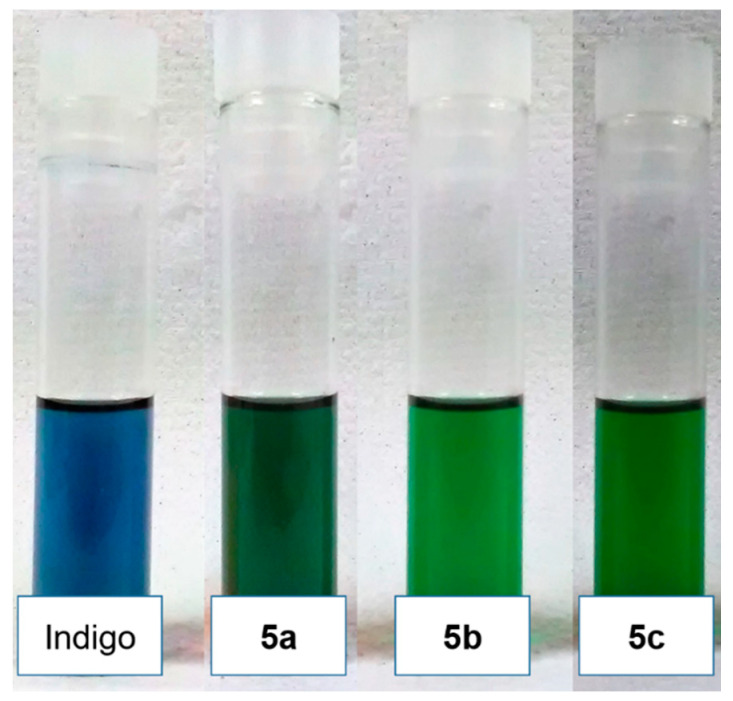
Solutions of dyes **5a** (2.3 × 10^−4^ M), **5b** (3.5 × 10^−4^ M) and **5c** (7.8 × 10^−5^ M) in CH_2_Cl_2_, in comparison with indigo suspension in CHCl_3_ (0,17 mg/mL ≈ 6.5 × 10^−4^ M).

In EtOH, unprotected dyes (especially compounds **5a** and **5c**) featured a much less intense and broadened spectrum, with a long tail extending in the near-IR region above 750 nm: this was attributed to their reduced solubility in that solvent, leading to the formation of aggregates and observation of light scattering effects. In addition, for most of the compounds, the lowest energy absorption peak was red-shifted in the more polar solvent, and in all cases, smaller *E_0-0_* values were recorded (1.82–1.87 eV): this bathochromic shift when the dielectric constant of the solvent is increased is well-known also for the parent indigo compound [47] and has been attributed to increased stability of charged-separated structures (for instance, C^+^–O^−^) in the excited state rather than in the ground state [9]. As a result of their light absorption profiles, compounds **5a**–**c** gave bright green to dark green-coloured solutions (Figure 6), which were different from the typical blue colour of indigo, demonstrating that the placement of donor moieties on 6,6′-positions of the main chromophore could significantly alter the optical properties of the resulting substances.

**Figure 7:**
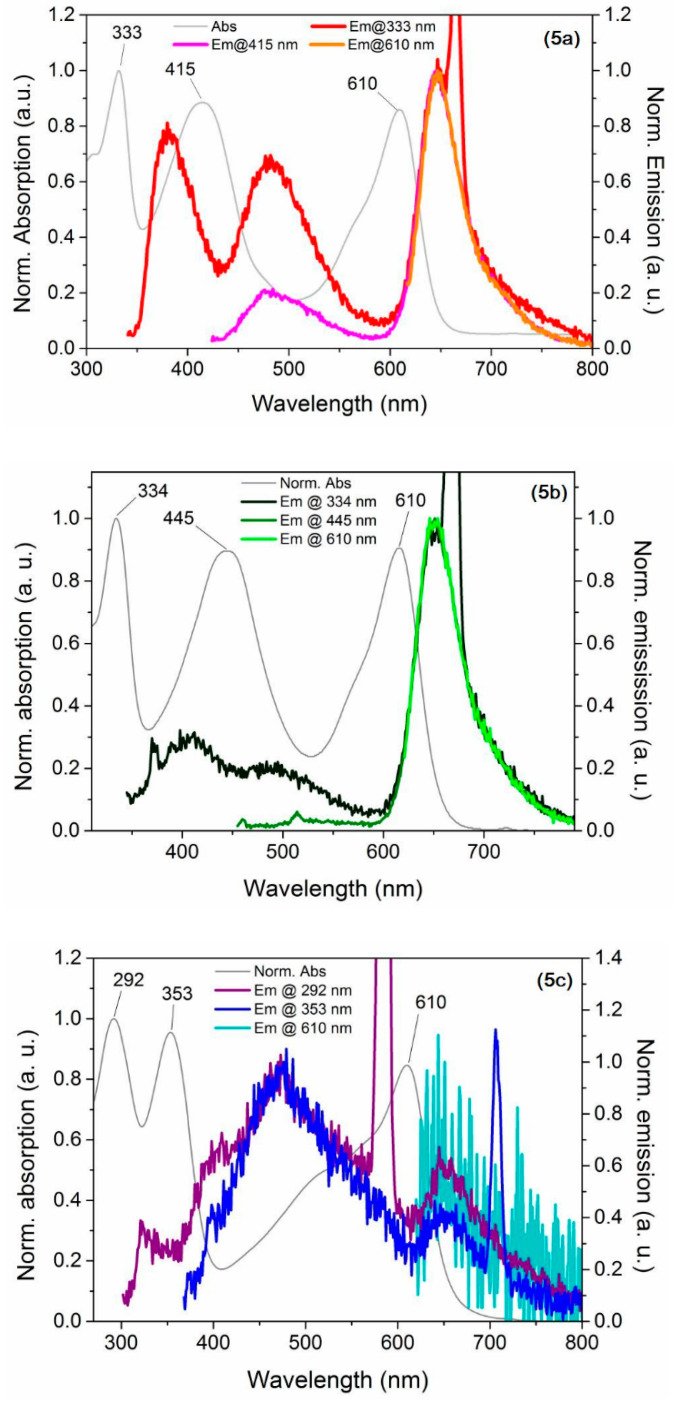
UV−Vis absorption and fluorescence emission spectra of compounds **5a**–**c** in CH_2_Cl_2_ solution. (**5a**): in the emission trace after excitation at 333 nm (red line) the peak visible at 666 nm is due to the second-order diffraction of the incident light. (**5b**): in the emission trace after excitation at 334 nm (dark green line) the sharp peak visible at 668 nm is due to the second-order diffraction of the incident light. (**5c**): in the emission traces after excitation at 292 nm (purple line) and 353 nm (blue line), the sharp peaks visible at 584 nm and 706 nm are due to the second-order diffraction of the incident light.

The fluorescence behavior of the new dyes was complicated by the possible occurrence of several different emissive transitions, as illustrated by the emission spectra obtained for compound **5a** at three different excitation wavelengths (Figure 7, top). After excitation at 333 nm (corresponding to an S_0_–S_3_ transition), three different fluorescence peaks were observed at 380, 481 and 647 nm, respectively (the latter was partially covered by a peak at 666 nm due to second-order diffraction of the incident radiation). While the first of them was due to the opposite S_3_–S_0_ transition, the other two likely originate from S_2_–S_0_ and S_1_–S_0_ transitions, demonstrating the possibility of non-radiative decay from the S_3_ state to the lower excited states of the dye. This was confirmed by the fact that irradiating at 415 nm only the peaks at 481 and 647 nm were observed, while only the latter was visible when irradiating at the wavelength of the indigo transition (610 nm).

In the case of compounds **5b** (Figure 7, middle) the situation was slightly different. Irradiating a solution of **5b** at 334 nm (thus populating the S_3_ state) induced only weak emissions at 411 and 493 nm, respectively, while a much more intense peak was observed at 646 nm, indicating that for this compound non-radiative decay to the S_1_ state was the preferred mean of energy dissipation when excited with higher energy radiation (also in this case a sharp peak at 668 nm was observed due to second-order diffraction of the incident radiation). This supposition was confirmed by the experiments conducted with irradiation at 445 and 610 nm, for which the lowest energy emission band at 646 was the only one observed.

The opposite behaviour was displayed by compound **5c** (Figure 7, bottom). In this instance, irradiation at 292 and 353 nm (whose absorption peaks should correspond to localized π→π* transitions within the triarylamine moiety) caused a fluorescence of moderate intensity centered at 474 nm, while only a very weak peak was seen at 645 nm. When irradiating at 610 nm, the fluorescence was only barely detectable, as shown by the very noisy normalized spectrum in Figure 7 indicating the prevalence of non-radiative decay from the lowest excited state S_1_ or the occurrence of extensive reabsorption by the dye. 

**Figure 8:**
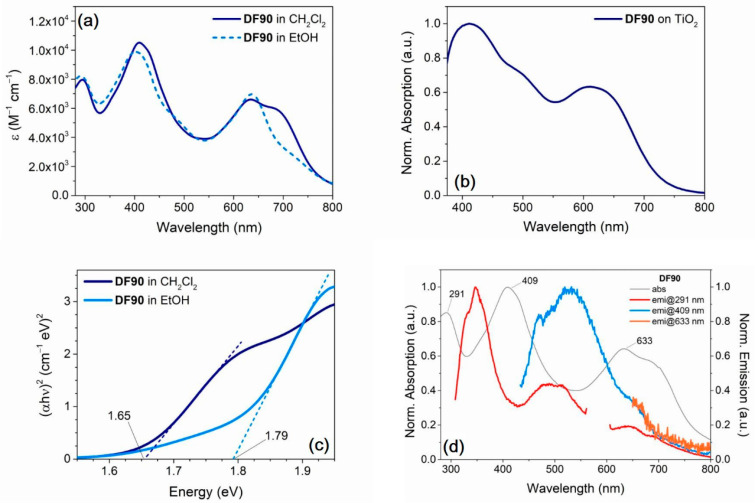
UV−vis absorption spectra of dye **DF90** in CH_2_Cl_2_ and EtOH solution (**a**), and adsorbed on TiO_2_ (**b**); Tauc plots corresponding to the absorption spectra in solution (**c**); fluorescence emission spectra in CH_2_Cl_2_ solution after excitation at different wavelengths (**d**); in the latter scheme, a peak at 582 nm deriving from second-order diffraction of incident light was deleted for clarity (red trace).

Finally, the spectra of the unsymmetrical D-A-π-A dye **DF90** (Figure 8) were registered. In solution, the dye exhibited absorption spectra similar to those of the symmetrical compounds, with a significantly red-shifted low energy transition (633–635 nm in CH_2_Cl_2_ and EtOH, respectively), whose molar extinction coefficient was however only moderate (0.65–0.70 × 10^4^ M^−1^ cm^−1^). The spectrum in CH_2_Cl_2_ also presented a shoulder at longer wavelengths relative to the indigo H-chromophore transition, which was attributed to the low solubility of **DF90** with the consequent formation of J (head to tail)-aggregates [49]. Accordingly, Tauc plots for both spectra resulted in estimated E_0-0_ values of 1.65–1.79 eV, which were smaller than those calculated for indigo and its symmetrical derivatives. When adsorbed on TiO_2_, **DF90** gave a very broad UV-Vis spectrum with the main peaks at 411 nm (shoulder at 495 nm) and 612 nm, and onset around 720–730 nm. The blue-shift of the spectrum compared to the one in solution could be due both to the formation of H (parallel)-aggregates as well as the deprotonation of the carboxylic function upon anchoring onto the semiconductor [50,51].

Due to the absorption minimum centered at about 550 nm, also in the case of **DF90** the resulting solutions as well as the semiconductor surface assumed a bright green coloration.

The emission behaviour of **DF90** in CH_2_Cl_2_ was qualitatively similar to that already observed for the symmetrical compound **5c**, with relatively strong fluorescence peaks corresponding to the S_3_-S_0_ and S_2_-S_0_ transitions, while the emission peak at approx. 650 nm was very weak (or even visible only as a shoulder of the more intense peak at 530 nm), perhaps due to extensive reabsorption by the wide absorption band between 600 and 750 nm. This is not surprising considering that both **5c** and **DF90** share the same donor group.

**Figure 9:**
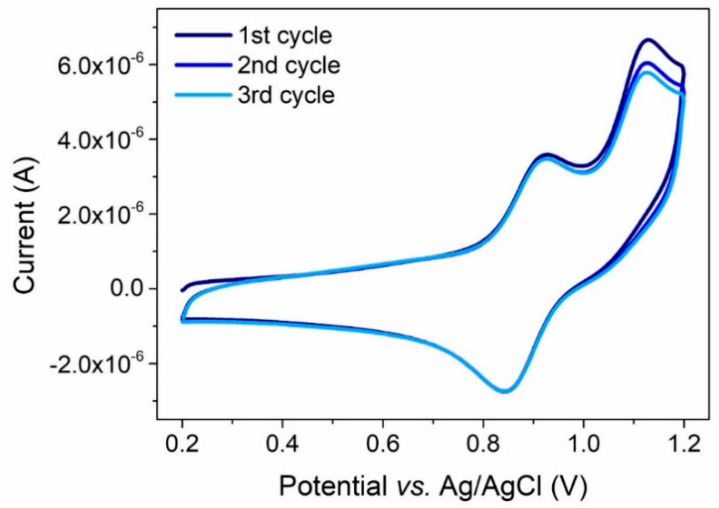
Cyclic Voltammetry plot relative to compound **DF90** in CH_2_Cl_2_ solution.

Finally, the ground-state oxidation potential (E_S+/S_) of **DF90** was measured by means of cyclic voltammetry (CV), which was carried out in THF and is reported in Figure 9.

The observed curve indicated a reversible oxidation process and the observed potential (0.89 V vs. Ag/AgCl/satd. KCl, corresponding to 0.69 V vs. NHE) was more positive than the redox potential of the iodide/triiodide couple (0.35 V vs NHE), ref. [52] suggesting that regeneration of the sensitizer during operation of a solar cell was possible.

After a small current drop following the first cycle (perhaps due to the consumption of the dye physisorbed on the glassy carbon surface), the current/voltage curve remained practically identical in the following two cycles, indicating that the dye was sufficiently stable upon repeated oxidation/reduction processes. The E_S+/S_ and E_0-0_ values in the same solvent were then used to calculate the excited state oxidation potential (E_S+/S*_) by means of the equation E_S+/S*_ = E_S+/S_ − E_0-0_. E_S+/S*_ was found to be around −0.96 V vs. NHE, thereby more negative than the conduction band edge of the semiconductor (−0.5 V vs NHE) [53] and therefore appropriate to allow electron injection from the excited state dye to titania.

## 3. Experimental Section

### 3.1. General Information

Unless otherwise stated, all reagents were purchased from commercial suppliers and used without purification. *t*-Boc-protected 6-6′-dibromoindigo (**11**) [38] and stannanes **12c [46]**, **12d** and **12e [44]** were prepared as previously reported. All air-sensitive reactions were performed using Schlenk techniques. Solvents used in cross-coupling reactions were previously degassed by means of the “freeze-pump-thaw” method. Tetrahydrofuran (THF) was freshly distilled immediately prior to use from sodium/benzophenone. CH_2_Cl_2_, toluene, and acetonitrile were dried on a resin exchange Solvent Purification System. Petroleum ether, unless specified, is the 40–70 °C boiling fraction. Organic phases derived from aqueous work-up were dried over Na_2_SO_4_. Reactions were monitored by TLC on SiO_2_ plates, the detection was made using a KMnO_4_ basic solution or UV lamp**.** Flash column chromatography was performed using glass columns (10–50 mm wide) and SiO_2_ (230–400 mesh). ^1^H-NMR spectra were recorded at 200, 300, or 400 MHz and ^13^C-NMR spectra at 50.0, 75.5, or 100.6 MHz, respectively. Chemical shifts were referenced to the residual solvent peak (CDCl_3_, δ 7.26 ppm for ^1^H-NMR and δ 77.16 ppm for ^13^C-NMR; THF-*d*_8_ δ 3.58 and 1.72 ppm for ^1^H-NMR, δ 67.21 and 25.31 ppm for ^13^C-NMR; CD_2_Cl_2,_ δ 5.32 ppm for ^1^H-NMR, δ 53.84 ppm for ^13^C-NMR). Coupling constants (*J*) are reported in Hz. ESI-MS were recorded with LCQ-Fleet Ion-Trap Mass Spectrometer. HR-MS were performed using an LTQ Orbitrap FT-MS Spectrometer. FT-IR spectra were recorded with a Perkin-Elmer Spectrum BX instrument in the range 4000–400 cm^−1^ with a 2 cm^−1^ resolution. UV-Vis spectra were recorded with a Varian Cary 400 spectrometer and a Shimadzu 2600 series spectrometer, and fluorescence spectra were recorded with a Varian Eclipse instrument, irradiating the sample at the wavelength corresponding to maximum absorption in the UV spectrum. UV-Vis spectra in different solvents were recorded on diluted solutions of the analyte (approximately 10^−5^ M) with a Shimadzu UV2600 spectrometer. UV-vis absorption or transmittance spectra of the compounds adsorbed on TiO_2_ were recorded in transmission mode after the sensitization of thin, transparent semiconductor films (thickness approximately 5 μm). 

Cyclic voltammetry experiments were conducted in chloroform solutions with a PARSTAT 2273 electrochemical workstation (Princeton Applied Research) employing a three-electrode cell having a 3 mm glassy carbon working electrode, a platinum counter electrode and an aqueous Ag/AgCl (sat. KCl) reference electrode and using ferrocene as a standard. The supporting electrolyte was electrochemical-grade 0.1 M [*N*(Bu)_4_]PF_6_; the dye concentration was approximately 10^−3^ M. Under these experimental conditions, the one-electron oxidation of ferrocene occurs at E^0′^ = 0.55 V.

### 3.2. Computational Details

Molecular and electronic properties of keto–keto (KK), keto–enol (KE) and enol-enol (EE) tautomers of compounds **5a**, **5b**, **5c**, **5d**, **6d** and **DF90** have been computed via DFT [54,55,56] and time-dependent DFT (TD-DFT) [57,58] methods, using the Gaussian 16, Revision B.01 suite of programs [59]. Geometry optimizations have been carried out *in vacuo* and in solvent (DCM) using the polarizable continuum model (PCM) [60] to take into account solvent effects, at the B3LYP/6-31G** level of theory [61,62], according to a previous work of Amat et al. [9]. For molecule **5b**, methyl groups have been used in place of the alkyl chains attached to the ProDOT moiety in order to reduce the computational cost. Frequency calculations on the optimized structures have been performed at the same level to check that the stationary points were true energy minima. The ground-state electron density delocalization and the energy of DFT frontier molecular orbitals have been calculated at the same level of theory in DCM. The UV-Vis spectroscopic properties of the analyzed compounds, including absorption maximum (λ^a^_max_), vertical excitation energy (E_exc_) and oscillator strengths (*f*) have been calculated in DCM on the minimized structures by means of TD-DFT at the B3LYP/6-311++G** and CAM-B3LYP/6-311++G** levels of theory [63]. 

### 3.3. Synthesis

#### 3.3.1. Synthesis of Tributyl(3,3-dipentyl-3,4-dihydro-2H-thieno[3,4-b][1,4]dioxepin-6-yl)-stannane (**12b**)

3,3-Dipentyl-3,4-dihydro-2*H*-thieno[3,4-b][1,4]dioxepine (ProDOT, 1.35 g, 4.56 mmol, 1.0 eq.) was dissolved in dry THF (14 mL). The solution was cooled to −78 °C, and *n*-BuLi (1.6 M solution in hexanes, 3.2 mL, 5.5 mmol, 1.2 eq.) was slowly added. The reaction mixture was allowed to warm up to −20 °C while stirring, then cooled down again to −78 °C. Tributyltin chloride (Bu_3_SnCl, 1.78 g, 5.5 mmol, 1.2 eq.) was added and the solution was allowed to warm up to room temperature and left under stirring overnight. The mixture was diluted with Et_2_O (20 mL) and washed with a cold saturated solution of NH_4_Cl (2 × 30 mL). The solvent was removed under reduced pressure to yield crude product **12b** (1.74 g, 3.0 mmol, 66% yield), which was used without further purification. ^1^H-NMR (200 MHz, CDCl_3_) δH = δH = 6.67 (1H, s), 3.75–3.87 (10H, m), 1.19–1.74 (24H, m), 0.76–1.03 (15H, m) ppm; ^13^C-NMR {^1^H} (50 MHz, CDCl_3_) δC = 155.7, 114.8, 111.0, 77.6, 43.8, 43.7, 32.7, 32.2, 29.0, 27.9, 27.2, 22.5, 14.0, 13.7, 10.7 ppm.

#### 3.3.2. General Procedure for Preparation of Compounds **6a**–**d**

Pd_2_(dba)_3_ (10 mg, 0.011 mmol, 0.05 eq.) and P(o-Tol)_3_ (7 mg, 0.022 mmol, 0.1 eq.) were dissolved in toluene (4 mL) and the solution was left at room temperature, under stirring for 15 min. *t*-Boc-protected 6-6′ dibromoindigo (**11**, 93 mg, 0.22 mmol, 1.0 eq.) was then added and the mixture stirred at room temperature for additional 15 min. The required stannane (0.46 mmol, 2.1 eq.) was dissolved in dry toluene (4 mL), and added to the reaction mixture, that was then warmed up to 50 °C, left under stirring and monitored by TLC. After 5 h, the mixture was cooled to room temperature, the solvent was removed by rotatory evaporation, and the crude was purified by flash column chromatography.

*(E)-(1,1′-di-tert-butyl 3,3′-dioxo-6,6′-bis(thiophen-2-yl)-1H,1′H,3H,3′H-[2,2′-biindolylidene-1,1′-dicarboxylate)* (**6a**). Compound **11** (92.5 mg, 0.22 mmol, 1.0 eq.) was reacted with tributyl(thiophen-2-yl)stannane **12a** (173 mg, 0.46 mmol, 2.1 eq.). Purification (petroleum ether:EtOAc gradient from 50:1 to 10:1) gave **6a** (119 mg, 0.19 mmol) as a purple red solid. Yield 86 %. Spectroscopic data were in agreement with those already reported.[45] ^1^H-NMR (400 MHz, CDCl_3_) δH = 8.32 (2H, s), 7.77 (2H, d, *J* = 7.9 Hz), 7.52 (2H, d, *J* = 3.5 Hz), 7.48 (2H, dd, *J*_1_ = 7.9Hz, *J*_2_ = 0.8Hz), 7.43 (2H, d, *J* = 5.0 Hz), 7.17–7.13 (m, 2H), 1.69 (18H, s). ^13^C-NMR{^1^H} (100 MHz, CDCl_3_) δC = 182.8, 150.0, 149.7, 143.6, 141.8, 128.7, 127.2, 125.6, 124.8, 122.0, 121,8, 114.0, 113.6, 84.8, 28.4. FT-IR (neat): ν = 3006 (w), 2956 (m), 2924 (m), 2854 (m), 1743 (s), 1670 (s), 1603 (s), 1579 (m) cm^−1^. MS (ESI) *m*/*z* 627.1 [M + 1]^+^.

*(E)-(1,1′-di-tert-butyl 6,6′-bis({3,3-dipentyl-2H,3H,4H-thieno[3,4-b][1,4]dioxepin-6-yl})-3,3′- dioxo-1H,1′H,3H,3′H-[2,2′-biindolylidene]-1,1′-dicarboxylate)* (**6b**). Compound **11** (92.5 mg, 0.22 mmol, 1.0 eq.) was reacted with stannane **12b** (271 mg, 0.46 mmol, 2.1 eq.). Purification (CH_2_Cl_2_: petroleum ether = 50:1) gave **6b** (155 mg, 0.15 mmol) as a purple red solid. Yield 67%. 8.29 (2 H, br. s), 7.72 (2H, d, J = 8.2 Hz)), 7.61 (2 H, d, J = 8.2 Hz), 6.53 (2H, s), 4.02 (4H, s), 3.93 (4H, s), 1.67 (18H, s), 1.46–1.39 (8H, m), 1.38–1.25 (26H, m), 0. 91(12H, t, J = 6.9 Hz) ppm. ^13^C-NMR{^1^H} (100 MHz, CDCl_3_) δC =182.4, 150.5, 149.3, 147.9, 141.0, 129.0, 128.4, 125.4, 125.0, 121.1, 113.8, 104.9, 84.3, 77.7(2C), 43.7, 32.6, 32.0, 28.2, 28.0, 22.5, 14.0. FT-IR (neat): ν = 2926 (w), 2857 (w), 1605 (s), 1439 (s), 1374 (w) cm^−1^. HRMS (ESI) m/z calculated for C_60_H_79_N_2_O_10_S_2_: 1051.5170. Found: 1051.5177 [M + 1]^+^.

*(E)-(1,1′-di-tert-butyl 6,6′-bis(4-{bis[4-(hexyloxy)phenyl]amino}phenyl)-3,3′-dioxo-1H,1′H, 3H,3′H-[2,2′-biindolylidene]-1,1′-dicarboxylate)* (**6c**). Compound **11** (130 mg, 0.2 mmol, 1.0 eq.) was reacted with stannane **12c** (308 mg, 0.42 mmol, 2.1 eq). Purification (petroleum ether: EtOAc gradient from 50:1 to 10:1) gave **6c** (210 mg, 0.16 mmol) as a purple solid. Yield 78 %. ^1^H-NMR (200 MHz, CDCl_3_) δH = 8.29 (2H, s), 7.62–7.91 (6H, m), 7.53 (4H, d, J = 8.8 Hz), 7.36–7.47 (6H, m), 7.13 (8H, d, J = 8.8 Hz), 7.00 (4H, d, J = 8.8 Hz), 6.88 (8H, d, J = 8.8 Hz), 3.96 (8H, t, J = 6.4 Hz), 1.72–1.79 (8H, m), 1.65 (18H, s), 1.56–1.30 (24H, s), 0.88–0.97 (12H, m) ppm. ^13^C-NMR{^1^H} (50 MHz, CDCl_3_) δC 182.9, 156.0, 149.9, 149.6, 148.7 143.4, 140.1, 131.1, 130.5, 129.0, 128.4, 128.1, 127.2, 125.5, 124.4, 119.7, 115.5, 84.2, 68.4, 31.7, 29.4, 28.2, 25.8, 22.6, 14.1. FT-IR (neat): ν = 2940 (m), 2929 (m), 2856 (m), 1733 (m), 1673 (s), 1589 (s), 1507 (s) cm^−1^. HRMS (ESI) m/z calculated for C_86_H_100_N_4_O_10_: 1348.7434. Found: 1348.7455 [M]^+^.

*(E)-(1,1′-di-tert-butyl 6,6′-bis(4-{bis[4-(hexylsulfanyl)phenyl]amino}phenyl)-3,3′-dioxo-1H, 1′H,3H,3′H-[2,2′-biindolylidene]-1,1′- dicarboxylate)* (**6d**). Compound **11** (93 mg, 0.22 mmol, 1.0 eq.) was reacted with stannane **12c** (353 mg, 0.46 mmol, 2.1 eq). Purification (petroleum ether:EtOAc gradient from 50:1 to 10:1) gave **6d** (235 mg, 0.15 mmol) as a purple red solid (yield 68%). Due to rapid decomposition, compound 6d, could not be fully characterized. MS (ESI) m/z 1576.0

#### 3.3.3. General Procedure for the Preparation of Compounds **5a**–**c**

Compounds **6a**–**c** were dissolved in a 1:1 mixture of CH_2_Cl_2_ and TFA (5 mL) and the solution was left under stirring for 4 h at room temperature. The solvent was removed by rotatory evaporation, and the crude was purified by washing with petroleum ether or by flash column chromatography.

*(E)-6,6′-bis(thiophen-2-yl)-1H,1′H,3H,3′H-[2,2′-biindolylidene]-3,3′-dione* (**5a**) [45]. Deprotection of compound **6a** (70 mg, 0.11 mmol), after crystallization from petroleum ether, gave **5a** (44 mg, 0.10 mmol) as a green amorphous solid. Yield 93%. Spectroscopic data were in agreement with those already reported. [45] ^1^H-NMR (400 MHz, THF-*d*_8_) δH = 9.95 (2H, s), 7.64 (2H, d, J = 8.0 Hz), 7.55 (2H, dd, *J*_1_ = 3.6 Hz, *J*_2_ = 1.0 Hz), 7.52 (2H, d, *J* = 0.7), 7.49 (2H, d, *J* = 4.7), 7.27 (2H, dd, *J*_1_ = 8.0Hz, *J*_2_ = 1.4Hz), 7.12 (2H, dd, *J*_1_ = 5.0Hz, *J*_2_ = 3.6 Hz). ^13^C-NMR{^1^H} (100 MHz, THF-*d*_8_) δC 187.7, 154.0, 144.4, 141.9, 129.0, 127.4, 125.7, 124.9, 122.4, 119.7, 118.4, 109.7. FT-IR (neat): ν = 3344 (m), 3302 (m), 2954 (w), 2920 (m), 2850 (m), 1631 (s), 1610 (s), 1582 (s) cm^−1^. MS (ESI) *m*/*z* 427, 2 [M + H]^+^.

*(E)-6,6′-bis({3,3-dipentyl-2H,3H,4H-thieno[3,4-b][1,4]dioxepin-6-yl})-1H,1′H,3H,3′H-[2,2′-bis-indolylidene]-3,3′-dione* (**5b**). After purification (petroleum ether:EtOAc gradient from 35:1 to 10:1, then pure EtOAc), deprotection of compound **6b** (105 mg, 0.10 mmol) gave **5b** (82 mg, 0.96 mmol) as a green amorphous solid. Yield 96%. ^1^H-NMR (200 MHz, CDCl_3_) δH = 9.17 (2H, br. s.), 7.61 (2H, d, *J* = 8.1 Hz), 7.48 (2H, s), 7.12 (2H, d, *J* = 8.1 Hz), 6.45 (2H, s), 3.95 (4H, s), 3.88 (4H, s), 1.17–1.43 (32H, m), 0.81–0.98 (12H, m) ppm. ^13^C-NMR{^1^H} (50 MHz, CDCl_3_) δC = 187.4, 152.2, 150.5, 147.9, 141.0, 124.4, 122.2, 120.6, 118.5,118.1, 109.3, 104.6, 77.5(2C), 65.8, 43.8, 32.7, 32.1, 22.5, 14.0 ppm. FT-IR (neat): ν = 3385 (w), 2954 (m), 2926 (m), 2857 (m), 1624 (m), 1604 (s), 1572 (m), 1438 (s) cm^−1^. HRMS (ESI) *m*/*z* calculated for C_50_H_62_N_2_O_6_S_2_: 851.4122. Found: 851.4145 [M + 1]^+^.

*(E)-6,6′-bis(4-{bis[4-(hexyloxy)phenyl]amino}phenyl)-1H,1′H,3H,3′H-[2,2′-biindolylidene]-3,3′-dione* (**5c**). After purification (petroleum ether:EtOAc gradient from 35:1 to 10:1, then pure EtOAc), deprotection of compound **6c** (60 mg, 0.045 mmol), gave **5c** (52 mg, 0.044 mmol) as a green amorphous solid. Yield 92%. ^1^H-NMR (200 MHz, CDCl_3_) δH = 9.03 (2H, br. s.), 7.68 (2 H, d, *J*= 8.2 Hz), 7.41 (4 H, d, *J* = 8.8Hz), 7.10–7.17 (4H, m), 7.07 (8H, d, *J* = 9.3 Hz), 6.94 (4H, d, *J* = 8.2 Hz), 6.84 (8H, d, *J* = 9.3), 3.94 (8H, t, *J* = 6.3Hz), 1.74–1.83 (8H, m), 1.43–1.52 (8H, m), 1.32–1.37 (16H, m), 0.90–0.94 (12H, m); ^13^C-NMR{^1^H} (50 MHz, CDCl_3_) δC = 187.9, 155.9, 152.5, 149.4, 148.7, 140.0, 130.8, 127.8, 127.1, 124.7, 122.2, 119.5, 118.2, 117.1, 115.35, 109.2, 68.2, 31.6, 29.3, 25.7, 22.6, 14.0. FT-IR (neat): ν = 3368 (w), 2952 (w), 2927 (m), 2857 (m), 1627 (m), 1594 (s), 1503 (s), 1444 (s) cm^−1^. HRMS (ESI) *m*/*z* calculated for C_76_H_84_N_4_O_6_: 1148.6391. Found: 1148.6415 [M]^+^.

#### 3.3.4. Synthesis of Dye **DF90**

*Synthesis of (E)-(1,1′-di-tert-butyl 6-bromo-6′-(5-formylthiophen-2-yl)-3,3′-dioxo-1H,1′H, 3H,3′H- [2,2′-biindolylidene]-1,1′-dicarboxylate)* (**13**). Pd_2_(dba)_3_·CHCl_3_ (86.0 mg, 0.08 mmol, 0.2 eq.) and P(o-Tol)_3_ (100.0 mg, 0.32 mmol, 0.7 eq.) were dissolved in toluene (150 mL) and the solution was left under stirring for 15 min. t-Boc-protected 6-6′-dibromoindigo (**11**, 1.50 g, 2.4 mmol, 5.0 eq.) was then added and the mixture stirred at room temperature for additional 15 min. A solution of 5-(tributylstannyl)thiophene-2-carbaldehyde 12e (190 mg, 0.48 mmol, 1.0 eq.) in toluene (10 mL) was added and the mixture warmed to 50 °C overnight. The solvent was then removed by rotatory evaporation and a mixture of EtOAc:hexane = 1:20 (100 mL) was added to the residue. The insoluble fraction was washed several times with the same mixture, allowing to recover starting material **11**. The organic phase, after evaporation, gave a crude solid which was purified by flash chromatography (CH_2_Cl_2_ then CH_2_Cl_2_:EtOAc 50:1) to afford aldehyde **13** (310 mg, 0.48 mmol) as a red amorphous solid. Yield 79%. ^1^H-NMR (200 MHz, CDCl_3_) δH = 9.95 (1H, s), 8.39 (1H, d, J = 1.5 Hz), 8.27 (1H, d, J = 1.5 Hz), 7.79–7.85 (2H, m), 7.57–7.65 (2H, m), 7.54 (1H, dd, J_1_ = 7.9, J_2_ = 1.5), 7.38 (1H, dd, J_1_ = 8.1, J_2_ = 1.5). 1.72 (9H, s), 1.69 (9H, s). ^13^C-NMR{^1^H} (50 MHz, CDCl_3_) δC = 182.7, 182.4, 182.3, 152.3, 149.6, 149.5, 149.4, 144.0, 140.1, 137.1, 131.0, 127.7, 126.1, 126.0, 125.0, 124.9, 124.8, 123.0, 122.5, 122.4, 121.7, 120.2, 114.4, 85.2, 85.1, 28.1, 28.0; MS (ESI) m/z 673.0 [M + Na]^+^.

S*ynthesis of di-tert-butyl (E)-6-(4-(bis(4-(hexyloxy)phenyl)amino)phenyl)-6′-(5-formylthiophen-2-yl)-3,3′-dioxo-[2,2′-biindolinylidene]-1,1′-dicarboxylate* (**14**). Pd_2_(dba)_3_·CHCl_3_ (6.0 mg, 0.02 mmol, 0.2 eq.) and P(o-Tol)_3_ (31.0 mg, 0.10 mmol, 0.7 eq.) were dissolved in THF (4 mL) and the solution was left under stirring for 15 min. Bromoaldehyde **13** (100.0 mg, 0.15 mmol, 1.0 eq.) was then added and the mixture stirred at room temperature for an additional 15 min. A solution of stannane **12c** (220.0 mg, 0.30 mmol, 2.0 eq) in THF (1 mL) was added to the mixture and warmed to 55 °C. After 7 h the solvent was removed by rotatory evaporation, the crude residue was dissolved with EtOAc (10 mL) and washed with an aqueous saturated KF solution, then with brine. The organic phase was dried, then removed under vacuum to give a crude product which was purified by flash chromatography (CH_2_Cl_2_: Petroleum ether = 1:1 then CH_2_Cl_2_: Ethyl acetate 20:1) to obtain **14** (93 mg, 0.15 mmol) as a red amorphous solid. Yield 61%. ^1^H-NMR (400 MHz, CD_2_Cl_2_) δH 9.95 (1H, s), 8.42 (1H, s), 8.24 (1H, s), 7.83–7.74 (m, 3H), 7.63 (1H, d, J = 3.9Hz) 7.58–7.53 (3H, m), 7.53–7.47 (m, 3H), 7.46 (1H, dd, J_1_ = 7.8Hz, J_2_ = 1.5Hz m), 7.12 (4H, d, J = 9.0Hz), 6.98 (2H, d, J = 9.0Hz), 6.89 (4H, d, J = 9.0Hz), 3.97 (4H, d, J = 6.4Hz), 1.82–1.75 (4H, m), 1.66 (9H, s), 1.63 (9H, s), 1.55–1.45 (4H, m), 1.40–1.32 (8H, m), 0.92 (6H, t, J = 5.9 Hz); ^13^C-NMR{^1^H} (100 MHz, CD_2_Cl_2_) δC = 182.8, 182.7, 182.3, 152.1, 149.9, 149.8, 149.7, 149.3, 148.7, 144.0, 139.8, 139.7, 137.2, 130.4, 127.9, 127.3, 126.2, 126.1, 124.7, 124.6, 124.3, 123.2, 122.2, 122.1, 120.8, 119.2, 115.3, 114.1, 113.5, 84.7, 84.4, 68.3, 31.6, 29.3, 27.8, 25.7, 22.6, 13.8; MS (ESI) Found: m/z 1015.25 [M]^+^.

*Synthesis of (2E,Z)-3-{5-[(E)-6′-(4-{bis[4-(hexyloxy)phenyl]amino}phenyl)-3,3′-dioxo-1H,1′H, 3H,3′H-[2,2′-biindolyliden]-6-yl]thiophen-2-yl}-2-cyanoprop-2-enoic acid* (**DF90**).To a solution of aldehyde **14** (95 mg, 0.093 mmol, 1 eq) in a mixture of acetonitrile/CH_2_Cl_2_ (1:1, 4 mL, dried on molecular sieves) were added cyanoacetic acid (10 mg, 0.12 mmol, 1.3 eq) and dry piperidine (84 mg, 0.99 mmol, 8 eq). The mixture was refluxed for 1 h, then it was cooled and the solvent removed under vacuum. The crude was dissolved in CH_2_Cl_2_ (10 mL) and washed with NaOH 0.1M and finally with HCl 0.1M. The organic phase was dried on Na_2_SO_4_, then the solvent was removed under vacuum. The crude product was recrystallized from CH_2_Cl_2_/ethyl acetate (2:1) to obtain **DF90** (45 mg, 0.093 mmol) as a green solid. Yield 55%. ^1^H-NMR (400 MHz, THF-d_8_) δH = 9.99 (2H, br s), 8.39 (1H, s), 7.89 (1H, d, J = 3.9 Hz), 7.70–7.62 (4H, m), 7.52 (2H, d, J = 9.0 Hz), 7.42 (1H, s), 7.38 (1H, d, J = 8.2 Hz), 7.20 (1H, d, J = 8.6 Hz), 7.08–7.05 (4H, m), 6.93 (2H, d, J = 9.0 Hz), 6.88-6.85 (4H, m), 3.95, (4H, t, J = 6.4 Hz), 1.81–1.74 (4H, m), 1.53–1.46 (4H, m), 1.39–1.35 (8H, m), 0.93 (6H, t, J = 7.0 Hz); ^13^C-NMR{^1^H} (100 MHz, CDCl_3_) δC= 188.2, 187.6, 163.4, 157.2, 154.5, 153.8, 153.3, 150.6, 149.2, 146.5, 141.2, 139.9, 137.5, 132.2, 128.5, 128.0, 126.8, 125.2, 124.9, 123.3, 122.2, 121.2, 120.4, 119.5, 119.1, 118.6, 116.5, 116.1, 111.5, 111.1, 110.9, 110.4, 68.8, 32.6, 30.4, 26.8, 23.6, 14.4; FT-IR (neat): ν = 2923 (w), 2853 (m), 1704 (m), 1586 (s), 1505 (s), 1445 (s) cm^−1^. HRMS (ESI) m/z calculated for C_54_H_50_N_4_O_6_S: 882,3451. Found: 882.3435 [M]^+^.

## 4. Conclusions

In this paper, a mild synthetic approach to prepare indigo based dyes is reported. Tyrian Purple is used as starting material to be coupled, after *t*-Boc protection, with different stannanes under Stille-Migita conditions. Three new symmetrical D-A-D dyes featuring an extended conjugation have been prepared and spectroscopically characterized and their optical properties compared with the results of theoretical calculations. Furthermore, non-symmetrical indigo-based D-A-π-A dye has been designed, synthesized and fully characterized both spectroscopically and electrochemically, assessing the possibility of using the indigo scaffold to prepare green dyes for DSSC application

## Figures and Tables

**Table 1:** TD-DFT (B3LYP/6-311++G**) absorption maxima (λ^a^max), excitation energies (E_exc_), oscillator strengths (f) and contribution (%) to the transition in DCM for the lowest excited states having a non-negligible oscillator strength of KK tautomer of compounds **5a**, **5b**, **5c**, **5d** and **DF90**.

Molecule	Excited States	λ^a^max (nm)	E_exc_ (eV)	f	Contribution (%)
**5a**	1	604	2.05	0.54	100% H→L
**5b**	1	610	2.03	0.58	100% H→L
**5c**	1	760	1.63	0.86	99% H→L
	3	594	2.08	0.32	99% H-2→L
**5d**	1	746	1.62	0.76	99% H→L
	3	596	2.08	0.36	99% H-2→L
**DF90**	1	857	1.44	0.32	99% H→L
	2	645	1.92	0.41	97% H-1→L
**DF90 ^#^**	1	543	2.28	0.66	80% H-1→L

^#^ computed at the CAM-B3LYP/6-311++G** level of theory.

**Table 2:** Optical properties of dyes **5a**–**d** and **6a**–**c** compared with those of parent indigo compound.

Dye	*λ_max_* CH_2_Cl_2_ [nm]	ε CH_2_Cl_2_ [× 10^4^ M^−1^ cm^−1^]	*E_0-0_* CH_2_Cl_2_ [eV] *^a^*	*λ_max_* EtOH [nm]	ε EtOH [× 10^4^ M^−1^ cm^−1^]	*E_0-0_* EtOH [eV] *^a^*
**Indigo**	604 *^b^*	0.93 *^b^*	1.96 *^b^*	-	-	-
**6a**	324	1.75	2.14	323	1.74	2.14
	406	2.04		411	1.94	
	546	0.41		545	0.3	
**5a**	333	1.14	1.95	333	0.52	1.85
	415	1.01		414	0.44	
	610	1		620	0.38	
**6b**	328	2.4	2.11	- *^c^*	- *^c^*	- *^c^*
	426	1.7				
	548	0.9				
**5b**	335	3.05	1.94	335	3.06	1.87
	445	2.77		441	2.56	
	614	2.88		626	2.57	
**6c**	290	5.5	2.06	296	4.52	2.03
	346	5.15		347	5.2	
	554	2.9		556	2.88	
**5c**	293	4.7	1.93	293	3.2	1.82
	355	4.55		351	2.89	
	610	4.06		608	1.93	
**6d**	324	6.4	2.1	325	6.89	2.09
	548	1.11		549	1.09	

*^a^ E_0-0_* of the lowest energy transition, estimated by means of the corresponding Tauc plot (see Figure S6). *^b^* Spectrum recorded in CHCl_3_. *^c^* The UV-Vis absorption spectrum of this compound could not be recorded in EtOH due to insufficient solubility.

## Supplementary Materials

The following is the Supplementary data to this article: computational details for compounds **5a**, **5b**, **5c**, **5d**, **6d**, and **DF90**; Tauc plots for the CH_2_Cl_2_ solutions of compounds **3a–d** and **4a–c** and for for the EtOH solutions of compounds **3a**,**c**,**d** and **4a–c**; copies of the ^1^H and ^13^C NMR spectra of compounds **5a, 5b**, **5c** and **DF90.**
