# Supplementary material for: Synthesis and Characterization of New Organic Dyes Containing the Indigo Core
Source: Molecules. 2020 Jul 25;25(15):3377. doi: 10.3390/molecules25153377 (PMC7435895; doi:10.3390/molecules25153377)

Supporting Information

Synthesis and Characterization of New Organic Dyes Containing the Indigo Core

Daniele Franchi [1,2,3], Massimo Calamante [1,2], Carmen Coppola [4,5], Alessandro Mordini [1,2,*], Gianna Reginato [2,*], Adalgisa Sinicropi [2,4,5] and Lorenzo Zani [2]

1 Dipartimento di Chimica "Ugo Schiff", Università degli Studi di Firenze, Via della Lastruccia, 13, 50019 Sesto Fiorentino, Italy; daniele.franchi87@gmail.com (D.F.); mcalamante@iccom.cnr.it (M.C.)
2 CNR-Istituto di Chimica dei Composti Organometallici (CNR-ICCOM), Via Madonna del Piano, 10, 50019 Sesto Fiorentino, Italy; adalgisa.sinicropi@unisi.it (A.S.); lorenzo.zani@iccom.cnr.it (L.Z.)
3 Department of Chemistry, KTH, Teknikringen 30, 10044 Stockholm, Sweden
4 R²ES Lab, Dipartimento di Biotecnologie, Chimica e Farmacia, Università degli Studi di Siena, Via A. Moro, 2, 53100 Siena, Italy; carmen.coppola@student.unisi.it
5 CSGI, Consorzio per lo Sviluppo dei Sistemi a Grande Interfase, Via della Lastruccia, 3, 50019 Sesto Fiorentino, Italy
* Correspondence: alessandro.mordini@iccom.cnr.it (A.M.); gianna.reginato@iccom.cnr.it (G.R.)

Table of Contents

1. Computational details for compounds **5a**, **5b**, **5c**, **5d**, **6d** and **DF90**…………………S2
2. Tauc plots for compounds **6a–d** and **5a–c** ………………………………………… …S13
3. Copies of the ^{1}H and ^{13}C NMR spectra of compounds **5a, 5b**, **5c** and **DF90** ……......S15

Computational Details

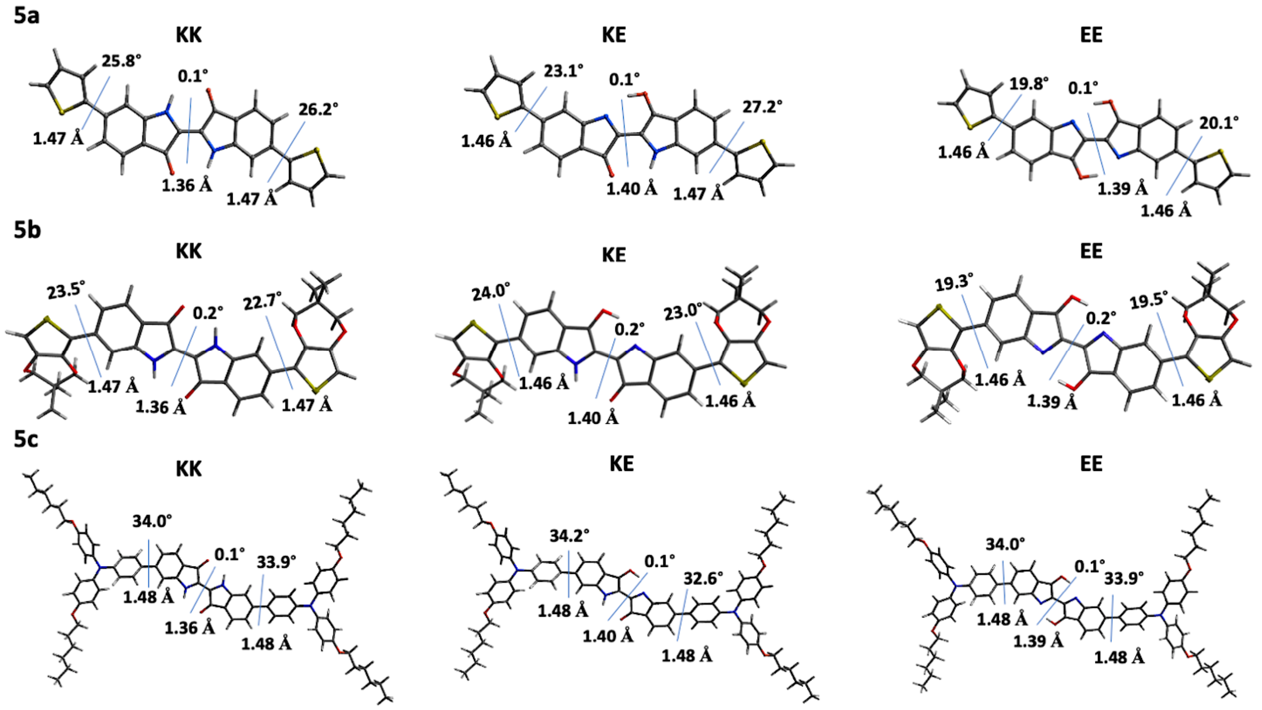

Figure S1. B3LYP/6-31G** optimized geometries in vacuo of KK, KE and EE tautomers of compounds **5a**, **5b** and **5c**.

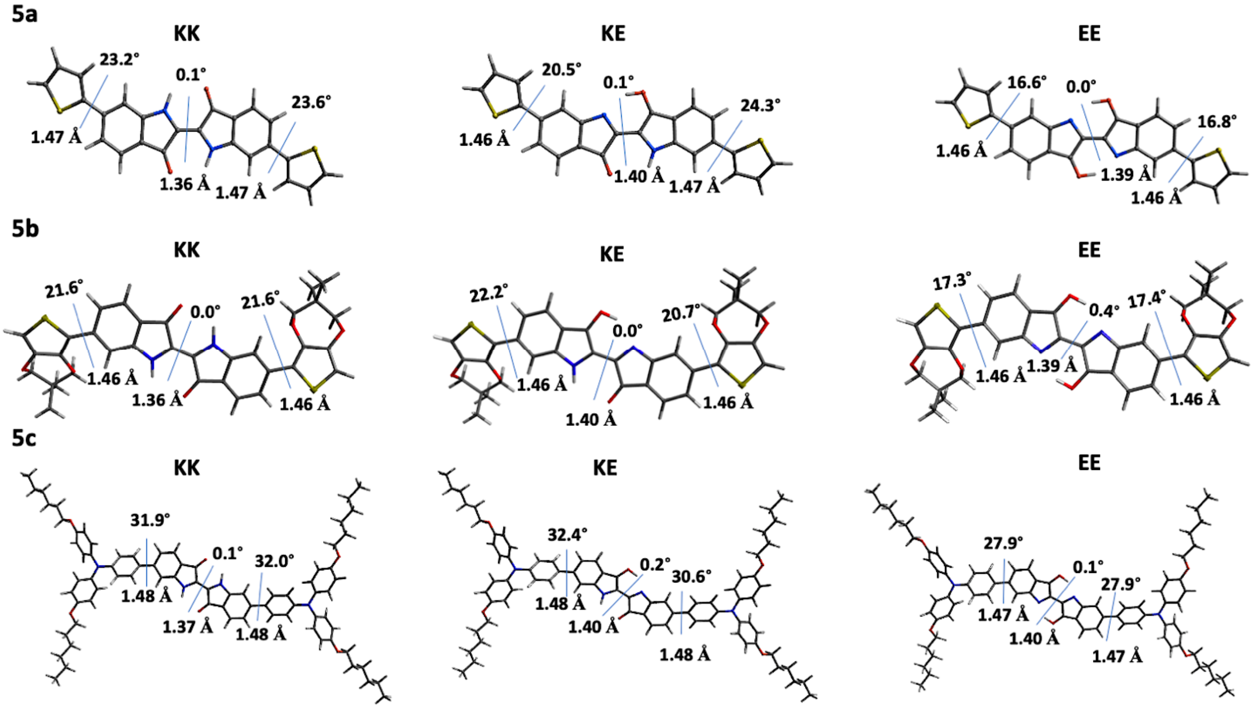

Figure S2. B3LYP/6-31G** optimized geometries in DCM of KK, KE and EE tautomers of compounds of **5a**, **5b** and **5c**.

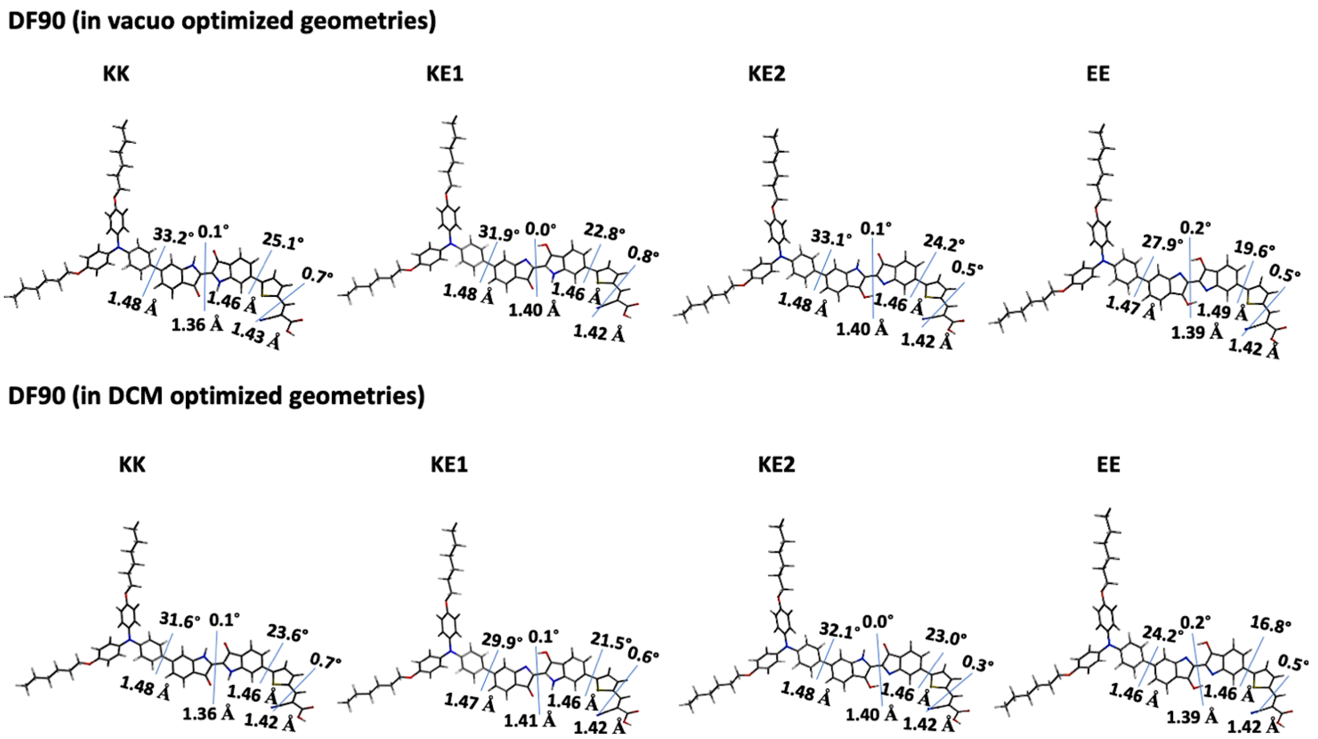

Figure S3. B3LYP/6-31G** optimized geometries in vacuo and in DCM of KK, KE and EE tautomers of compound **DF90**.

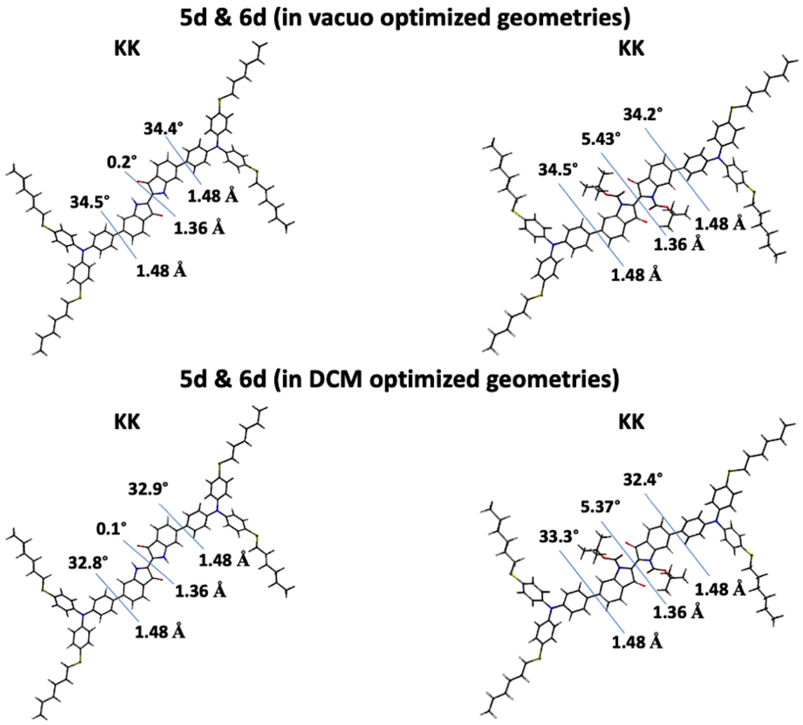

Figure S4. B3LYP/6-31G** optimized geometries in vacuo and in DCM of KK tautomer of compounds **5d** and **6d**.

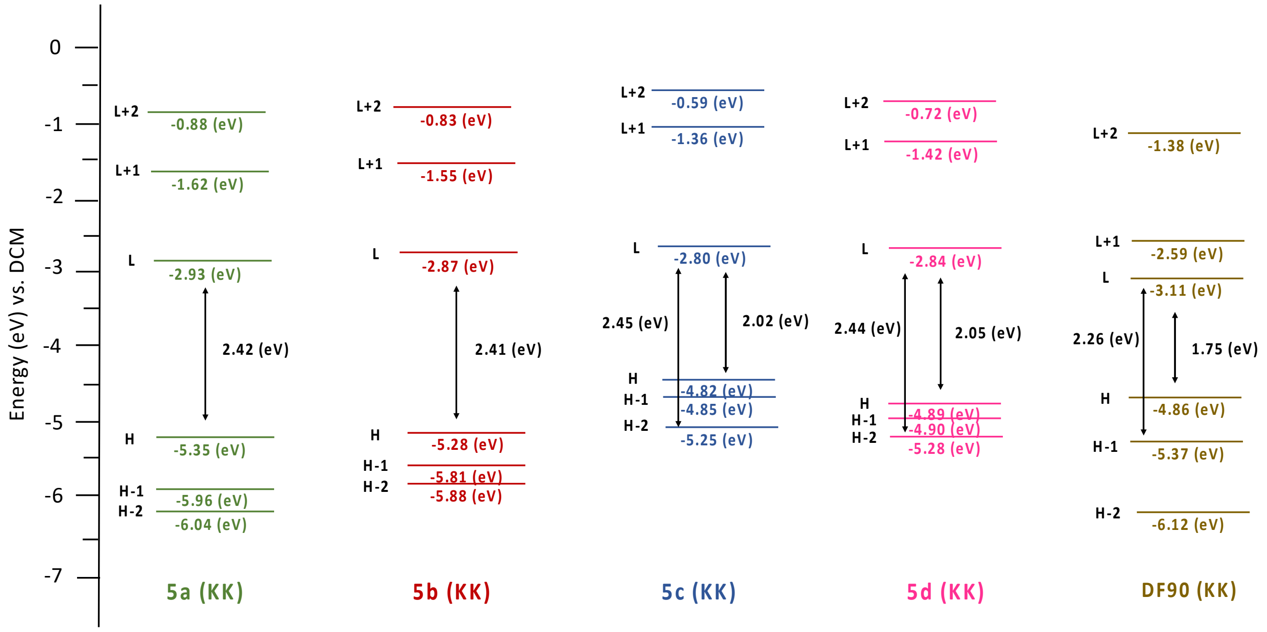

Figure S5. B3LYP/6-31G** FMOs energies in DCM solution of tautomer KK of **5a**, **5b**, **5c**, **5d** and **DF90**.

Table S1. B3LYP/6-31G** absolute and relative energies (kcal/mol) of KK, KE and EE tautomers of compounds **5a**, **5b**, **5c** and **DF90** computed *in vacuo* and DCM.

Molecule	Tautomer	Absolute energy in vacuo	ΔE	Absolute energy in DCM	ΔE
**5a**	KK	-1676035.507	0.00	-1676045.021	0.00
	KE	-1676025.916	+9.60	-1676034.216	+10.80
	EE	-1676002.483	+33.02	-1676010.483	+34.53
**5b**	KK	-1242060.895	0.00	-1242068.158	0.00
	KE	-1242051.572	+9.32	-1242057.493	+10.66
	EE	-1242028.210	+32.67	-1242033.516	+34.64
**5c**	KK	-2269806.326	0.00	-2269820.066	0.00
	KE	-2269796.788	+9.54	-2269808.638	+11.43
	EE	-2269773.874	+32.45	-2269785.628	+34.44
**DF90**	KK	-1980710.268	0.00	-1980725.508	0.00
	KE1	-1980701.877	+8.31	-1980715.832	+9.68
	KE2	-1980700.304	+9.96	-1980714.280	+11.23
	EE	-1980678.398	+31.87	-1980692.148	+33.36

Table S2. B3LYP/6-31G** absolute and relative[a] stabilities (kcal/mol) of KK, KE and EE tautomers of compounds **5a** and **DF90** in vacuo.

Molecule	Tautomer	Absolute stability	Relative stability
**5a**	KK	-1241896.059	0.0
	KE	-1241886.569	+9.49
	EE	-1241863.825	+32.23
**DF90**	KK	-1980229.065	0.0
	KE1	-1980220.368	+8.70
	KE2	-1980219.076	+9.99
	EE	-1980197.583	+31.48

[a]The Gibbs free energy of the most stable tautomer is taken as the reference (0.0 kcal/mol).

Table S3. B3LYP/6-31G* orbital energies of KK, KE and EE tautomers of compounds **5a**, **5b**, **5c** and **DF90** computed in DCM.

Molecule	Tautomer	HOMO-2	HOMO-1	HOMO	LUMO	LUMO+1	LUMO+2	Δ(H-L)
**5a**	KK	-6,045	-5,962	-5,350	-2,932	-1,620	-0,885	2.418
	KE	-6,089	-6,026	-5,290	-3,124	-1,587	-0,895	2.166
	EE	-6,137	-5,851	-5,072	-3,674	-1,588	-0,958	1.398
**5b**	KK	-5.875	-5.814	-5.276	-2.871	-1.547	-0.835	2.405
	KE	-5.960	-5.880	-5.201	-3.063	-1.505	-0.843	2.138
	EE	-5.987	-5.758	-4.990	-3.593	-1.484	-0.897	1.397
**5c**	KK	-5,253	-4,847	-4,824	-2,799	-1,355	-0.593	2.025
	KE	-5,312	-4,862	-4,757	-2,989	-1,333	-0,600	1.768
	EE	-5,148	-4,966	-4,707	-3,479	-1,291	-0,618	1.228
**DF90**	KK	-6,119	-5,374	-4,863	-3,114	-2,593	-1,382	1.749
	KE1	-6,165	-5,395	-4,894	-3,190	-2,647	-1,369	1.704
	KE2	-6,114	-5,405	-4,796	-3,304	-2,639	-1,378	1.492
	EE	-5,837	-5,224	-4,943	-3,704	-2,641	-1,345	1.239

Table S4. B3LYP/6-31G* orbital energies of KK tautomer of compounds **5d** and **6d** in DCM.

Molecule	Tautomer	HOMO-2	HOMO-1	HOMO	LUMO	LUMO+1	LUMO+2	Δ(H-L)
**5d**	KK	-5.282	-4.902	-4.885	-2.836	-1.416	-0.719	2.049
**6d**	KK	-5.909	-4.918	-4.906	-2.926	-1.615	-0.838	1.980

Table S5. TD-DFT ($: CAM-B3LYP/6-311++G** and #: B3LYP/6-311++G**) absorption maxima ($\lambda^{a}{}_{max}$), excitation energies (E_{exc}), oscillator strenghts (f) and contribution (%) to the transition in DCM of KK, KE and EE tautomers of compound **5a**.

**Molecule**	**Tautomer**	**Excited States**		λ^{a}**max (nm)**		E_{exc} **(eV)**		**f**		**Contribution (%)**	
		$	#	$	#	$	#	$	#	$	#
**5a**	**KK**	1	1	532.68	604.24	2.32	2.05	0.54	0.54	98%<br>H→L	100%<br>H→L
		4	3	363.03	473.55	3.41	2.61	1.46	0.71	82%<br>H-2→L	99%<br>H-2→L
		8	10	286.89	330.10	4.32	3.75	0.29	0.82	30%<br>H-7→L<br>5%<br>H-2→L<br>4%<br>H-2→L+6	81%<br>H-1→L+1
	**KE**	1	1	593.70	704.80	2.08	1.76	0.45	0.45	89%<br>H→L	99%<br>H→L
		2	6	423.34	383.71	2.93	3.23	0.25	0.66	69%<br>H-1→L	97%<br>H→L+1
		4	9	378.27	338.88	3.27	3.65	0.90	0.09	66%<br>H-2→L	86%<br>H-7→L
		5	11	339.62	312.15	3.65	3.97	0.20	0.39	34%<br>H-3→L<br>8%<br>H-2→L+1<br>4%<br>H→L	43%<br>H-1→L+1<br>40%<br>H→L+2
		6		312.79		3.96		0.36		58%<br>H→L+1	
	**EE**	1	1	972.54	1042.0<br>7	1.27	1.19	0.52	0.46	95%<br>H→L	100%<br>H→L
		3	3	489.64	599.36	2.53	2.06	1.49	1.10	85%<br>H-2→L	96%<br>H-2→L
		11	13	288.21	334.82	4.30	3.70	0.18	0.17	43%<br>H-1→L+1	64%<br>H-9→L
			14		329.10		3.76		0.78	15%<br>H→L+6	56%<br>H-1→L+1

Table S6. TD-DFT ($: CAM-B3LYP/6-311++G** and #: B3LYP/6-311++G**) absorption maxima (λ^{a}max), excitation energies (E_{exc}), oscillator strenghts (f) and contribution (%) to the transition in DCM of KK, KE and EE tautomers of compound **5b**.

**Molecule**	**Tautomer**	**Excited States**		**λ^{a}max (nm)**		**E_{exc} (eV)**		**f**		**Contribution (%)**	
		$	#	$	#	$	#	$	#	$	#
**5b**	**KK**	1	1	537.19	609.29	2.30	2.03	0.55	0.58	98% H→L	100% H→L
		3	3	375.43	495.28	3.30	2.50	0.14	0.70	79% H-6→L	100% H-2→L
		4	10	374.21	334.63	3.31	3.70	1.33	0.83	75% H-2→L	84% H-1→L+1
	**KE**	1	1	600.60	715.12	2.06	1.73	0.46	0.48	88% H→L	99% H→L
		2	3	430.28	518.36	2.88	2.39	0.36	0.39	66% H-1→L	81% H-2→L
		4	8	385.36	382.34	3.21	3.24	0.81	0.68	56% H-2→L	96% H→L+1
		5	12	344.76	314.21	3.59	3.94	0.15	0.28	23% H-4→L 19% H→L+1	40% H-8→L 4% H-9→L
		6		314.69		3.93		0.46		65% H→L+1	
	**EE**	1	1	975.26	1044.77	1.27	1.18	0.55	0.51	95% H→L	100% H→L
			3		622.60		1.99		1.10		96% H-1→L
		3	9	502.66	392.89	2.47	3.15	1.51	0.09	84% H-2→L	94% H-6→L
		6	16	359.71	328.22	3.44	3.77	0.03	0.99	82% H-5→L	73% H-1→L+1

Table S7. TD-DFT ($: CAM-B3LYP/6-311++G** and #: B3LYP/6-311++G**) absorption maxima (λ^{a}_{max}), excitation energies (E_{exc}), oscillator strenghts (f) and contribution (%) to the transition in DCM of KK, KE and EE tautomers of compound **5c**.

Molecule	Tautomer	Excited States		λ^{a}max (nm)		E_{exc} (eV)		f		Contribution (%)	
		$	#	$	#	$	#	$	#	$	#
**5c**	**KK**	1	1	532.61	759.60	2.32	1.63	0.72	0.86	90%<br>H-1→L	99%<br>H→L
			3		593.75		2.08		0.32		99%<br>H-2→L
		3	18	426.02	333.56	2.91	3.71	1.33	0.51	74%<br>H→L	58%<br>H→L+4
		9	24	311.70	316.43	3.97	3.91	1.48	0.21	27%<br>H-1→L+1<br>17%<br>H→L	28%<br>H→L+7<br>18%<br>H→L+7
		15	25	280.55	316.40	4.41	3.91	0.32	0.26	10%<br>H-1→L+13<br>7%<br>H-1→L+10	26%<br>H-1→L+7<br>18%<br>H→L+8
	**KE**	1	1	599.40	865.60	2.06	1.43	0.56	0.77	62%<br>H-2→L	92%<br>H→L
			3		653.93		1.89		0.16		98%<br>H-2→L
		6	11	329.57	400.65	3.76	3.09	1.39	0.24	26%<br>H→L+1<br>7%<br>H-4→L<br>7%<br>H→L	84%<br>H-1→L+1
		11		305.25		4.06		0.37		15%<br>H-1→L<br>15%<br>H-14→L	
			28		315.22		3.93		0.22		88%<br>H→L+6
		13		282.75		4.38		0.33		23%<br>H→L+13<br>12%<br>H→L+18	
	**EE**	1	1	981.33	1205.09	1.26	1.02	0.80	1.51	62%<br>H→L	100%<br>H→L
		3	3	597.41	903.99	2.07	1.37	1.38	0.16	48%<br>H-2→L	100%<br>H-2→L
			5		514.58		2.40		0.53	34%<br>H→L	51%<br>H-4→L
		5	24	403.68	345.30	3.07	3.59	0.48	0.65	51%<br>H-4→L	64%<br>H→L+2<br>66%<br>H→L+3<br>86%<br>H→L+4
		14	29	308.08	320.16	4.02	3.87	1.05	0.46	37%<br>H-1→L+1<br>8%<br>H-1→L+7	62%<br>H-3→L+1

Table S8 (to be continued). TD-DFT ($: CAM-B3LYP/6-311++G** and #: B3LYP/6-311++G**) absorption maxima (λ^{a}_{max}), excitation energies (E_{exc}), oscillator strenghts (f) and contribution (%) to the transition in DCM of KK, KE and EE tautomers of compound **DF90**.

**Molecule**	**Tautomer**	**Excited States**		**λ^{a}max (nm)**		**E_{exc} (eV)**		**f**		**Contribution (%)**	
		$	#	$	#	$	#	$	#	$	#
**DF90**	**KK**	1	1	542.90	856.55	2.28	1.44	0.66	0.32	80% H-1→L	99% H→L
			2		645.14		1.92		0.41		97% H-1→L
		3	10	413.86	405.06	2.99	3.06	1.03	0.42	67% H-2→L	78% H→L+2
		12	26	297.69	315.36	4.16	3.93	0.23	0.23	22% H→L+1<br>9% H→L+4<br>8% H-1→L+2	92% H→L+8
	**KE1**	1	1	582.82	881.81	2.12	1.40	0.55	0.43	84% H-1→L	99% H→L
			2		691.58		1.79		0.62		97% H-1→L
		4	10	405.74	406.29	3.05	3.05	0.72	0.38	55% H-1→L+1	86% H-2→L+1
			11		399.63		3.10		0.17		88% H→L+2
		11	25	300.23	314.00	4.12	3.95	0.40	0.22	26% H→L+1<br>21% H→L+2	93% H→L+7

Table S8 (continued). TD-DFT ($: CAM-B3LYP/6-311++G** and #: B3LYP/6-311++G**) absorption maxima (λ^{a}max), excitation energies (E_{exc}), oscillator strenghts (f) and contribution (%) to the transition in DCM of KK, KE and EE tautomers of compound **DF90**.

Molecule	Tautomer	Excited States		λ^{a}max (nm)		E_{exc} (eV)		f		Contribution (%)	
		$	#	$	#	$	#	$	#	$	#
DF90	KE2	1	1	633.30	1019.03	1.96	1.21	0.57	0.42	56% H-1→L	100% H→L
			2		724.02		1.71		0.11		99% H-1→L
			3		651.60		1.90		0.18		98% H→L+1
		5	10	393.19	414.89	3.15	2.99	0.98	0.67	46% H-3→L	86% H→L+2
		11		307.49		4.03		0.22		21% H→L+1 7% H-9→L	
			25		318.42		3.89		0.22		84% H→L+7
		14		281.77		4.40		0.29		18% H→L+12 14% H→L+14	
	EE	1	1	981.47	1170.17	1.26	1.05	0.75	1.20	51% H→L	95% H→L
			2		944.89		1.31		0.18		92% H→L
		3	5	620.10	571.95	1.99	2.17	1.09	0.72	40% H-1→L 39% H→L	81% H-3→L
		4	9	457.18	449.22	2.71	2.76	0.84	0.29	54% H-3→L	96% H-2→L+1
		18	26	294.14	315.34	4.21	3.93	0.19	0.24	15% H-11→L 9% H→L+4 6% H-14→L	57% H-2→L+2
			28		309.56		4.00		0.15		80% H→L+6

Table S9. TD-DFT ($: CAM-B3LYP/6-311++G** and #: B3LYP/6-311++G**) absorption maxima (λ^{a}max), excitation energies (E_{exc}), oscillator strenghts (f) and contribution (%) to the transition in DCM of KK tautomer of compounds **5d** and **6d.**

Molecule	Tautomer	Excited States		λ^{a}max (nm)		E_{exc} (eV)		f		Contribution (%)	
		$	#	$	#	$	#	$	#	$	#
**5d**	**KK**	1	1	532.68	746.44	2.32	1.62	0.69	0.76	93% H-2→L	99% H→L
		3	3	419.97	595.84	2.95	2.08	1.35	0.36	74% H→L	99% H-2→L
		7	7	315.62	436.81	3.92	2.83	1.45	0.62	20% H-1→L+1	93% H-6→L
			10		410.25		3.02		0.50	2% H-1→L+3	91% H-6→L
		12	16	305.43	345.48	4.05	3.58	0.60	0.51	32% H-6→L 17% H-1→L+1	47% H-8→L
		13	20	299.98	339.94	4.13	3.64	0.21	0.18	40% H→L+5 29% H-1→L+6	42% H→L+5 15% H-1→L+6
		14	21	299.89	339.85	4.13	3.64	1.02	0.78	42% H→L+6 27% H-1→L+5	51% H→L+6 27% H-1→L+6
			22		338.85		3.65		0.11	38% H-16→L 12% H-7→L+1	45% H→L+2 13% H-8→L
**6d**	**KK**	1	1	429.29	775.72	2.88	1.59	1.05	0.59	70% H-4→L	99% H→L
			3		501.13		2.47		0.30		96% H-4→L
		7	15	317.14	349.88	3.90	3.54	1.31	0.71	25% H-1→L+1	60% H→L+2
			21		339.03		3.65		0.67		52% H→L+6

Spectroscopic and Electrochemical Measurements

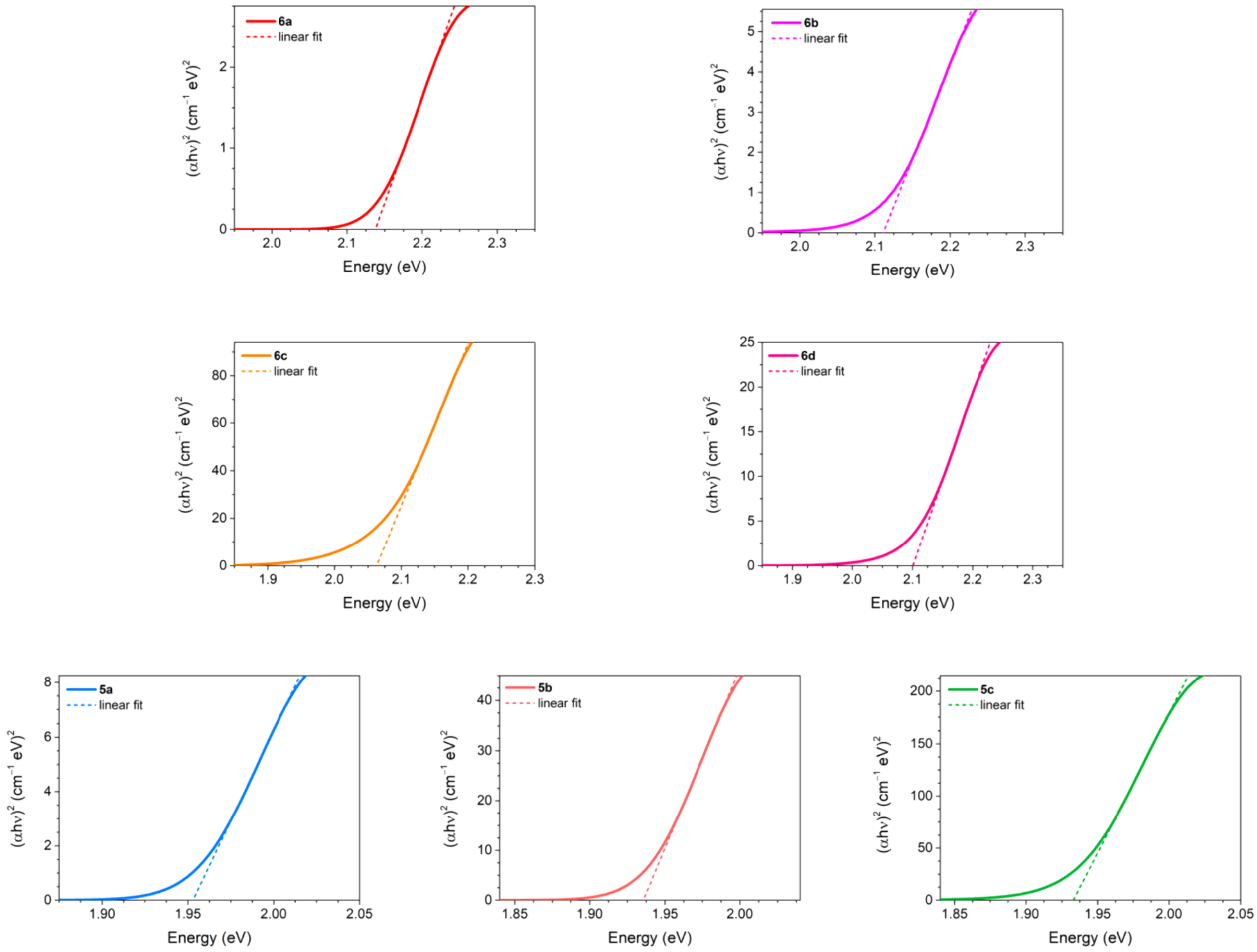

Figure S6. Tauc plots for the CH_2Cl_2 solutions of compounds **6a-d** and **5a-c**.

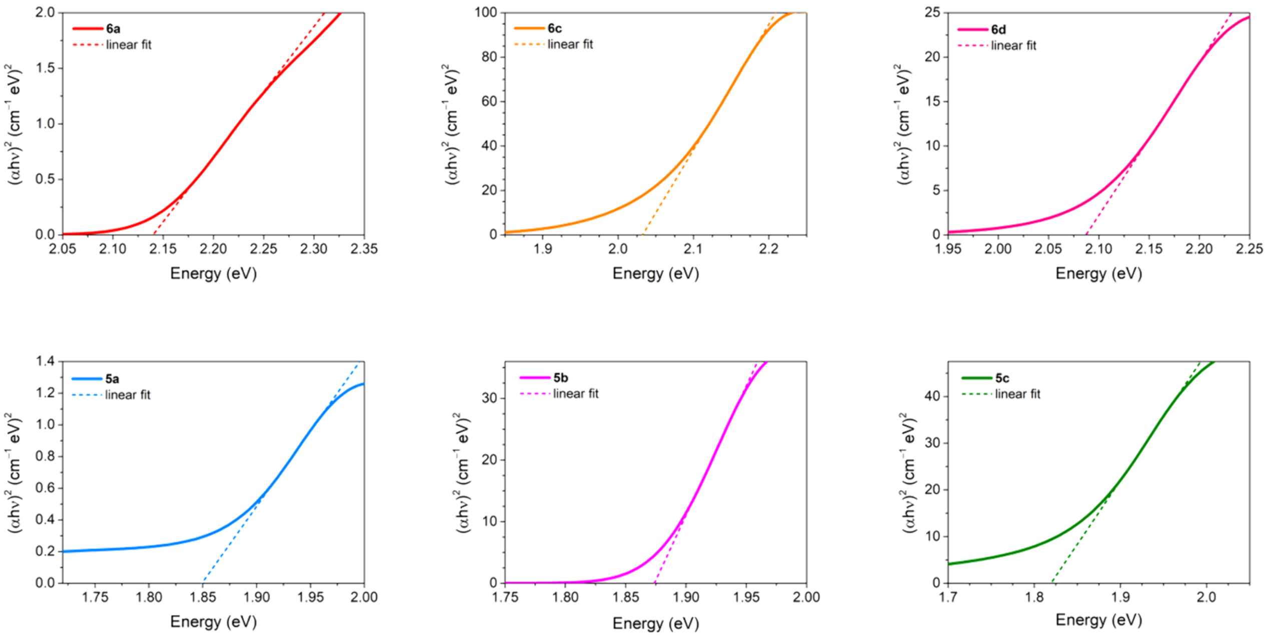

Figure S7. Tauc plots for the EtOH solutions of compounds **6a,c,d** and **5a-c**.

Copies of the NMR spectra of compounds 5a, 5b, 5c and DF90

Compound **5a**

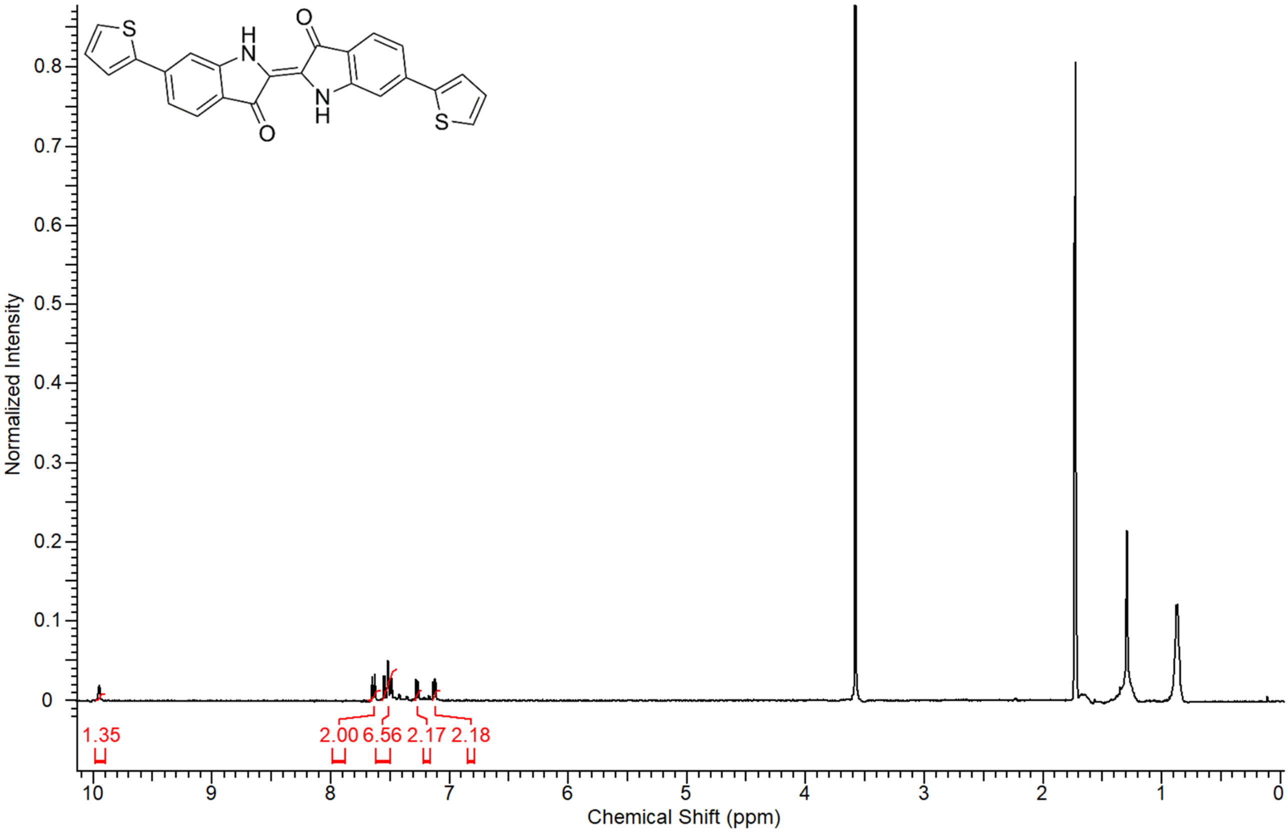

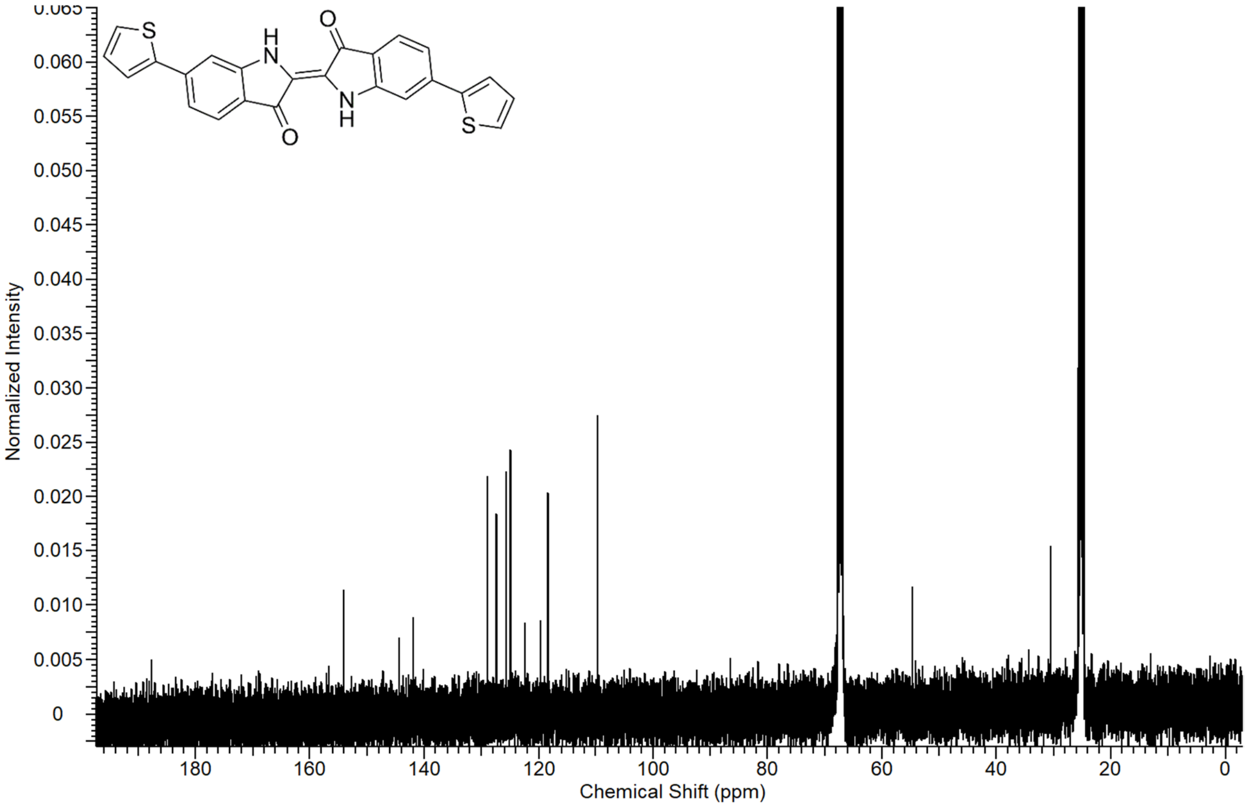

Compound **5b**

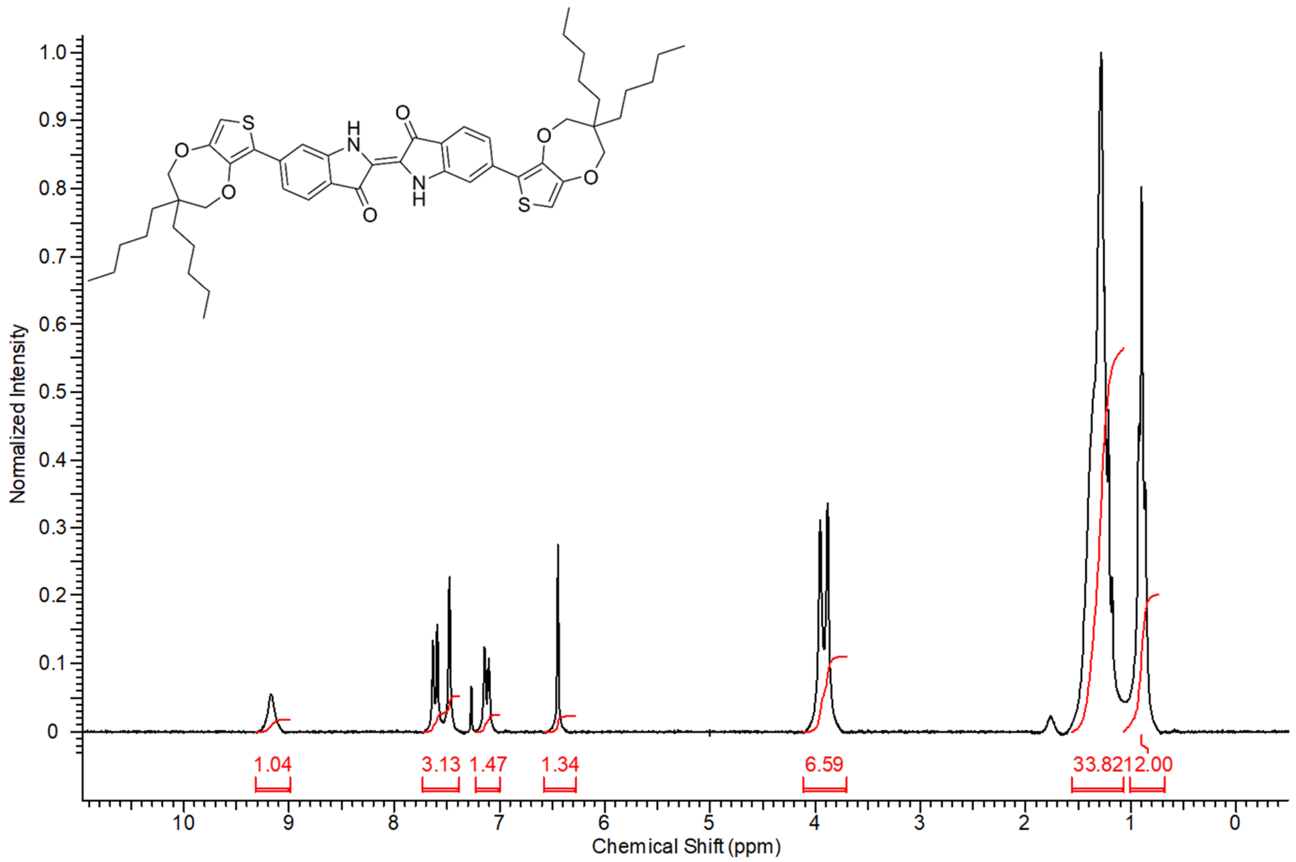

Normalized Intensity
Chemical Shift (ppm)
1.04
3.13
1.47
1.34
6.59
33.82
12.00

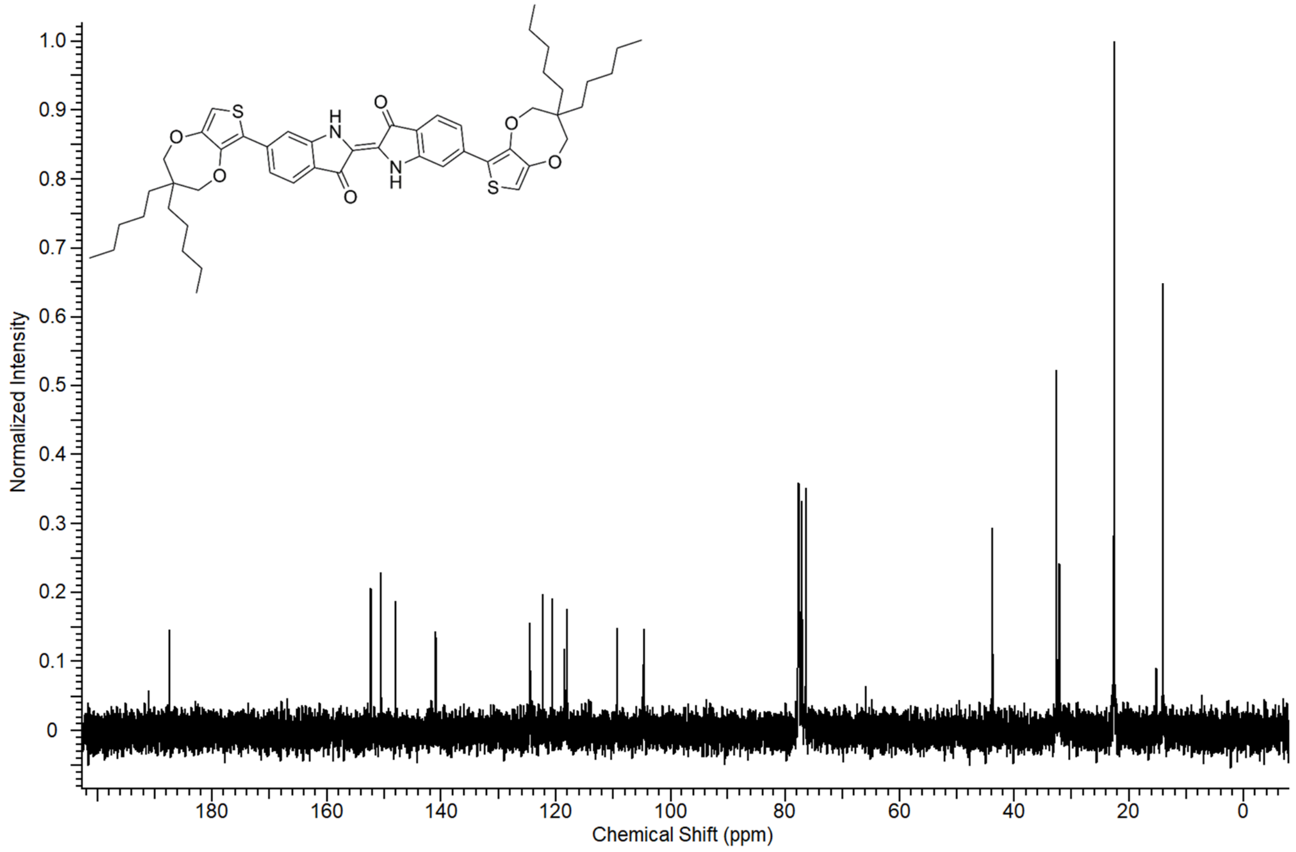

Normalized Intensity
Chemical Shift (ppm)

Compound **5c**

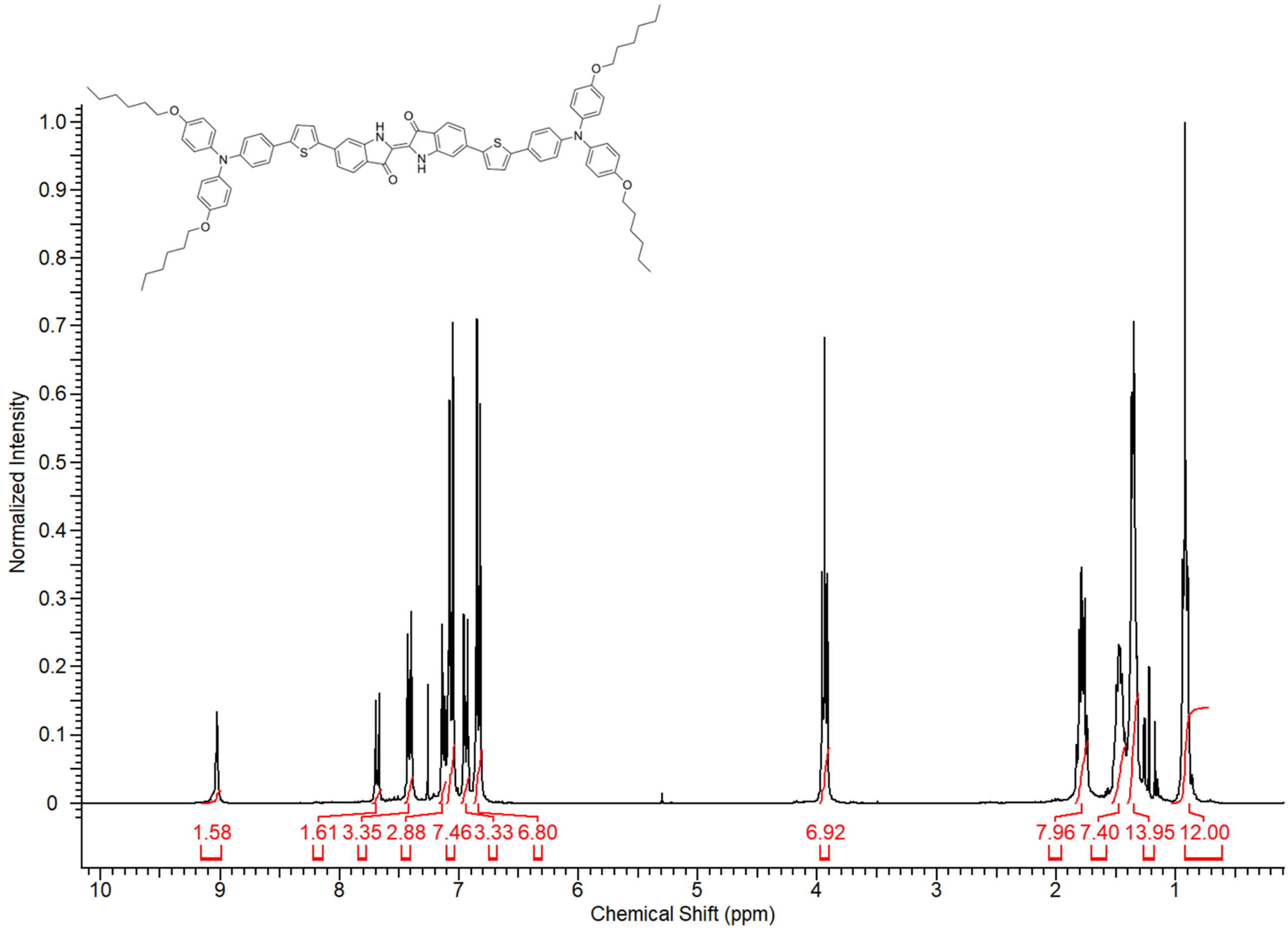
Normalized Intensity
Chemical Shift (ppm)
1.58 1.61 3.35 2.88 7.46 3.33 6.80
6.92
7.96 7.40 13.95 12.00

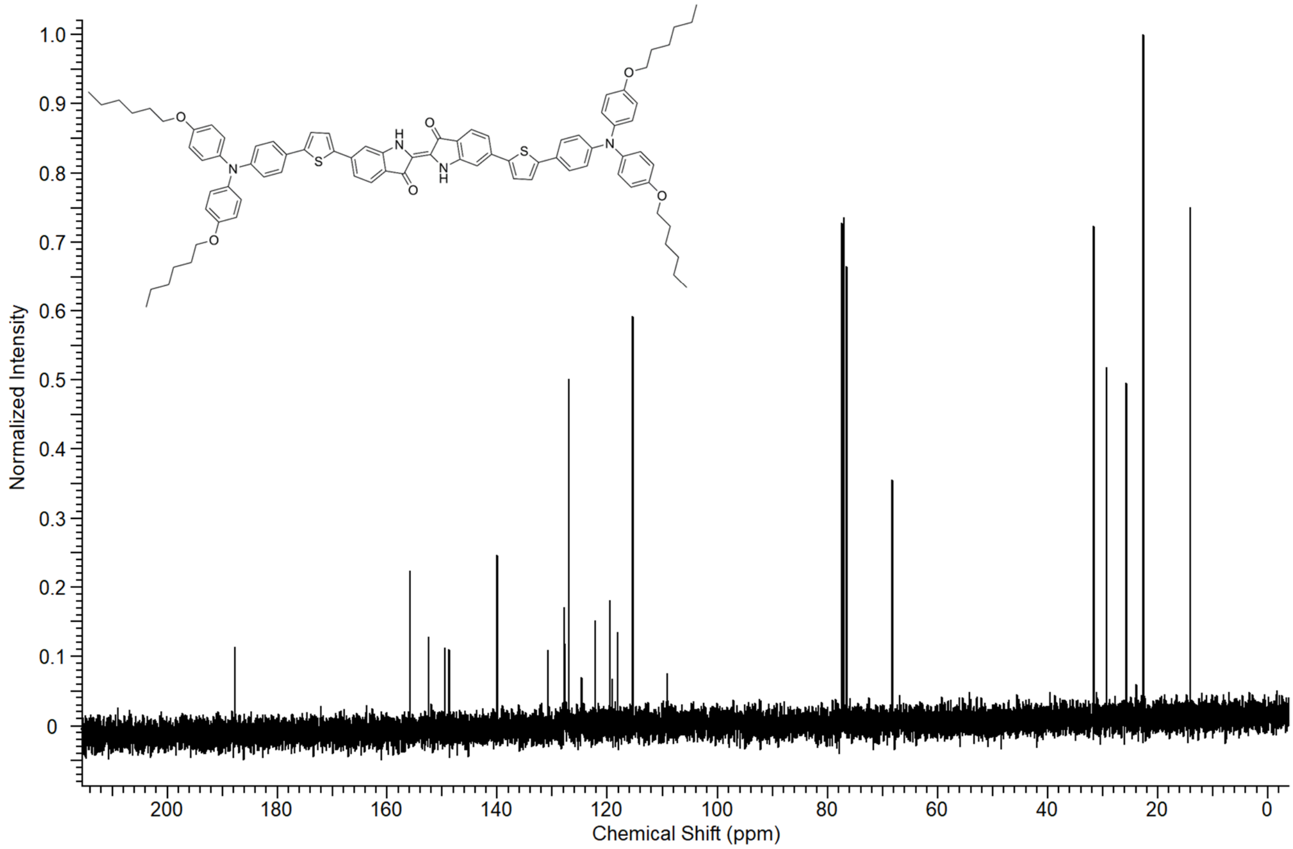
Normalized Intensity
Chemical Shift (ppm)

Compound **DF90**

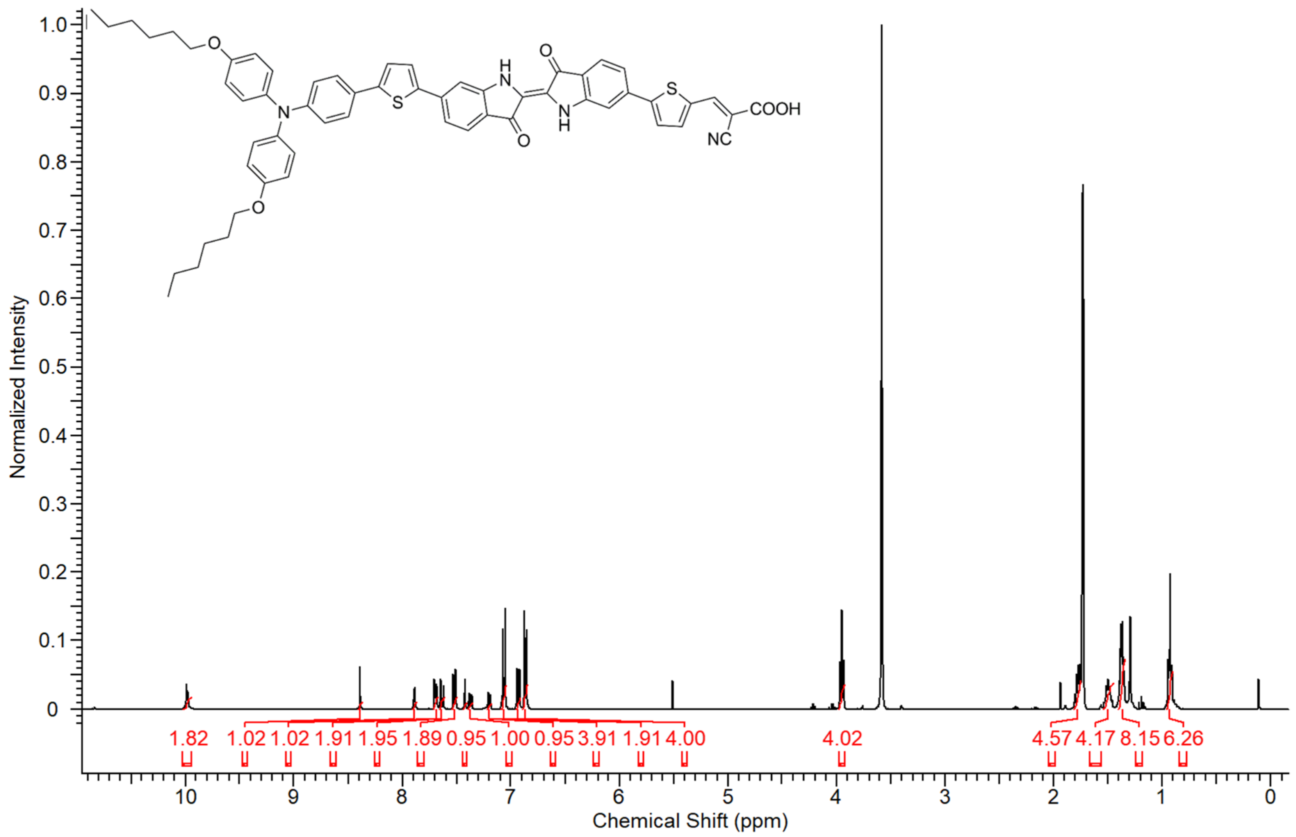

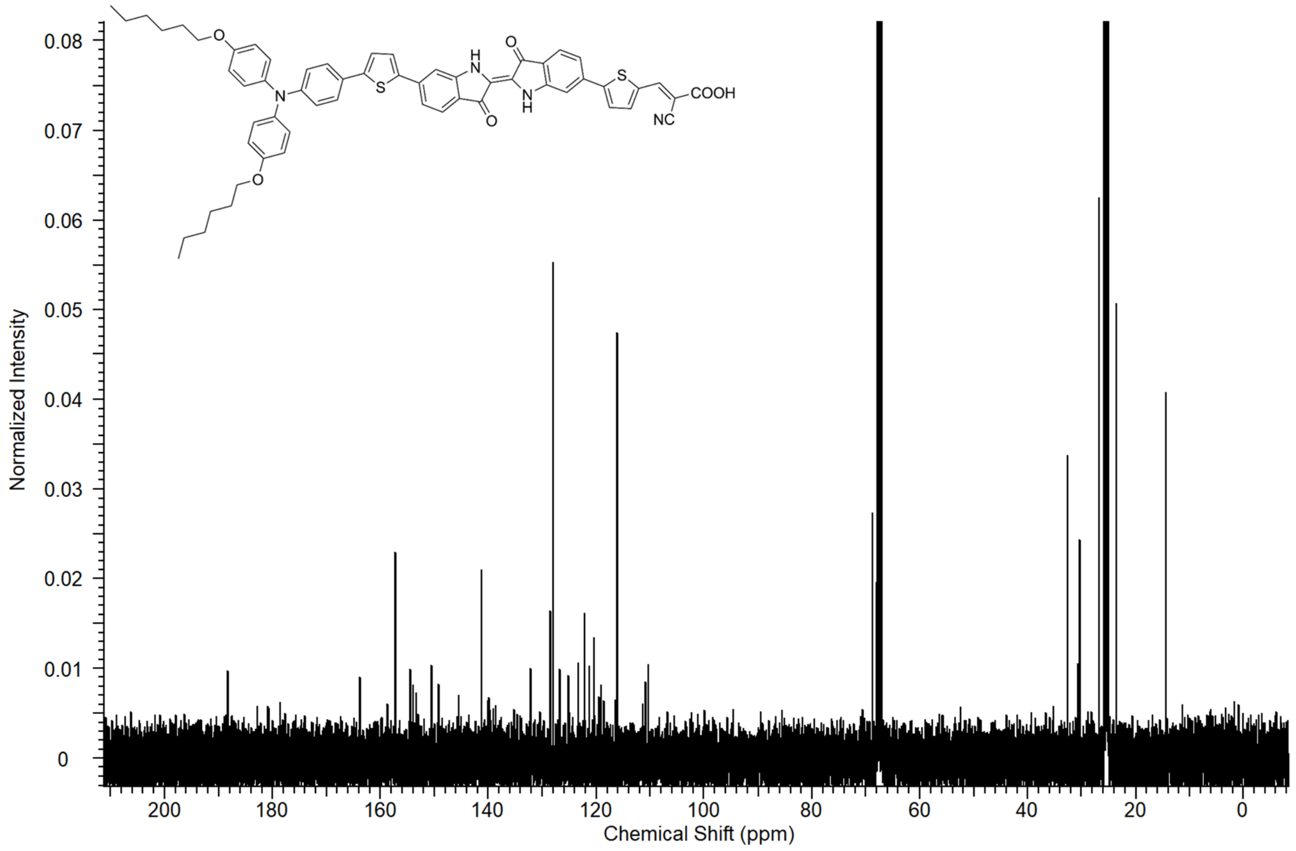

Supplement: Supplementary file 1 [file molecules-25-03377-s001.pdf]
